# Error-Reduced Digital Elevation Model of the Qinghai-Tibet Plateau using ICESat-2 and Fusion Model

**DOI:** 10.1038/s41597-024-03428-4

**Published:** 2024-06-05

**Authors:** Xingang Zhang, Shanchuan Guo, Bo Yuan, Haowei Mu, Zilong Xia, Pengfei Tang, Hong Fang, Zhuo Wang, Peijun Du

**Affiliations:** 1 Key Laboratory for Land Satellite Remote Sensing Applications of Ministry of Natural Resources, Jiangsu Provincial Key Laboratory of Geographic Information Science and Technology, School of Geography and Ocean Science, Nanjing University, Nanjing, China; 2 Jiangsu Center for Collaborative Innovation in Geographical Information Resource Development and Application, Nanjing, China; 3 School of Resource and Environmental Sciences, Wuhan University, Wuhan, China; 4 Faculty of Geomatics, Lanzhou Jiaotong University, Lanzhou, China

**Keywords:** Geomorphology, Geography, Geomorphology

## Abstract

The Qinghai-Tibet Plateau (QTP) holds significance for investigating Earth’s surface processes. However, due to rugged terrain, forest canopy, and snow accumulation, open-access Digital Elevation Models (DEMs) exhibit considerable noise, resulting in low accuracy and pronounced data inconsistency. Furthermore, the glacier regions within the QTP undergo substantial changes, necessitating updates. This study employs a fusion of open-access DEMs and high-accuracy photons from the Ice, Cloud, and land Elevation Satellite-2 (ICESat-2). Additionally, snow cover and canopy heights are considered, and an ensemble learning fusion model is presented to harness the complementary information in the multi-sensor elevation observations. This innovative approach results in the creation of HQTP30, the most accurate representation of the 2021 QTP terrain. Comparative analysis with high-resolution imagery, UAV-derived DEMs, control points, and ICESat-2 highlights the advantages of HQTP30. Notably, in non-glacier regions, HQTP30 achieved a Mean Absolute Error (MAE) of 0.71 m, while in glacier regions, it reduced the MAE by 4.35 m compared to the state-of-the-art Copernicus DEM (COPDEM), demonstrating its versatile applicability.

## Background & Summary

The Qinghai-Tibet Plateau (QTP), known as the “Roof of the World”^1^, is the largest and the highest plateau on the Earth, with an average elevation exceeding 4,000 m. This region is of critical importance in environmental and ecosystem research due to its profound impact on regional and global climate patterns. The Digital Elevation Model (DEM) is an indispensable dataset that depicts the surface terrain^2–4^, which characterizes and quantifies the intricate and varied terrain structure of the QTP. Ensuring the precise representation of the QTP’s terrain is of great importance for research in hydrology^5,6^, glaciology^7^, and geophysics^8^.

DEMs have long served as essential digital assets for studies in QTP. For example, the Advanced Spaceborne Thermal Emission and Reflection Radiometer Global DEM (ASTER GDEM)^9^ have been employed to assess the recent elevation increase at the QTP northeastern border^10^. The Advanced Land Observing Satellite (ALOS) World 3D - 30 m (AW3D30)^11^, Shuttle Radar Topography Mission (SRTM)^12^, NASADEM^13,14^, and TerraSAR-X add-on for Digital Elevation Measurement (TanDEM-X)^15,16^ have contributed to estimating QTP ice thickness^17^. The AW3D30 and SRTM have enabled the delineation of the QTP’s endorheic basins through automated method^18^. Furthermore, the Copernicus DEM (COPDEM)^19,20^ has supported studies on the QTP’s deformation^21^.

Nonetheless, generating accurate terrain representation for the QTP faces considerable challenges. Currently, none of the open-access DEMs can accurately and noise-free represent the terrain of the QTP^22–26^ (Supplementary Section 1). Stereo optical imagery-based^27,28^ DEMs and Synthetic Aperture Radar (SAR)-based DEMs^15,19,29,30^ are commonly employed worldwide, their accuracy within the QTP remains constrained, particularly in the high-altitude and rugged areas^31–33^. Specifically, speckle noise and stripe noise of SAR signals affect the accuracy of DEM^34,35^. For the optical image, the canopy in the forest region significantly affects the accuracy of the DEM^36^. The single surface texture of ice and snow regions makes stereo-matching more difficult. In addition, side-view observations in highly rugged regions can lead to missing data or limited expression, resulting in DEM voids and localized accuracy degradation. Furthermore, the QTP experiences substantial elevation fluctuations due to glacier retreat or advance^37,38^ and tectonic uplifts^39,40^, necessitating frequent DEM updates^26,41^.

The emergence of spaceborne Light Detection And Ranging (LiDAR) observations has opened up new avenues for acquiring high-accuracy elevation data, thereby enhancing DEM accuracy^42,43^. LiDAR altimetry generally provides higher elevation measurement accuracy than stereo photogrammetry and SAR-based techniques^44–46^. Notably, NASA launched the Ice, Cloud, and land Elevation Satellite-2 (ICESat-2) in September 2018, equipped with the Advanced Topographic Laser Altimeter System (ATLAS)^47^. This laser altimetry can acquire global-scale elevation data with a photon footprint diameter of 17 m^48^ and an anticipated vertical accuracy of 0.1 m^49^.

How to leverage this high-precision LiDAR data to construct accurate DEM? Researchers have utilized the high-density photons from ICESat-2 through interpolation to directly generate DEMs in Antarctica^50^ and Greenland^51^. Nevertheless, the sparse distribution, low density, and uneven sampling of ICESat-2 over the QTP prevent the direct generation of a continuous and complete DEM in QTP using interpolation methods. Other approaches involve using the ICESat-1 or ICESat-2 to correct existing DEMs through elevation assignments^52^, polynomial regression^36,53^, and multilayer perception methods^54^. However, these methods typically only correct individual DEM or consider elevation deviations using a few independent variables, without fully exploiting the complementary relationship between multiple DEMs and LiDAR altimetry.

Based on information fusion and ensemble learning theories, integrating complementary information from multi-sensor terrain observations, including LiDAR, SAR, and optical imagery, proves beneficial^55^. This study presents a novel high-accuracy and error-reduced 30 m DEM for the QTP (HQTP30), representing the 2021 QTP terrain. Data from ICESat-2 ATL06^41,56^, SRTM V3, newly published TanDEM-X 30 m Edited DEM (TAN30), as well as recorded glacier elevation changes since 2000^57^, were combined to obtain the 2021 QTP DEM for the glacier regions. Conversely, for the non-glacier regions, various high-precision data sources, including ICESat-2 ATL08^58,59^, AW3D30, COPDEM, TAN30, and NASADEM, were used to construct the QTP’s DEM in 2021. The derivation of HQTP30 relies on an Ensemble Learning Fusion (ELF) model, effectively harnessing the synergistic information within multiple DEMs and LiDAR data. Accuracy assessments of HQTP30 were conducted employing Google Earth Image, unmanned aerial vehicle (UAV)-derived DEMs, control points, and ICESat-2 data. HQTP30 is now publicly accessible through 10.6084/m9.figshare.24633927.v3, providing the scientific community and broader society with invaluable data for a range of scientific and societal applications, including the study of hydrological cycles, glacier dynamics, tectonic activity, and land surface processes within the QTP.

## Methods

**Fig. 1:**
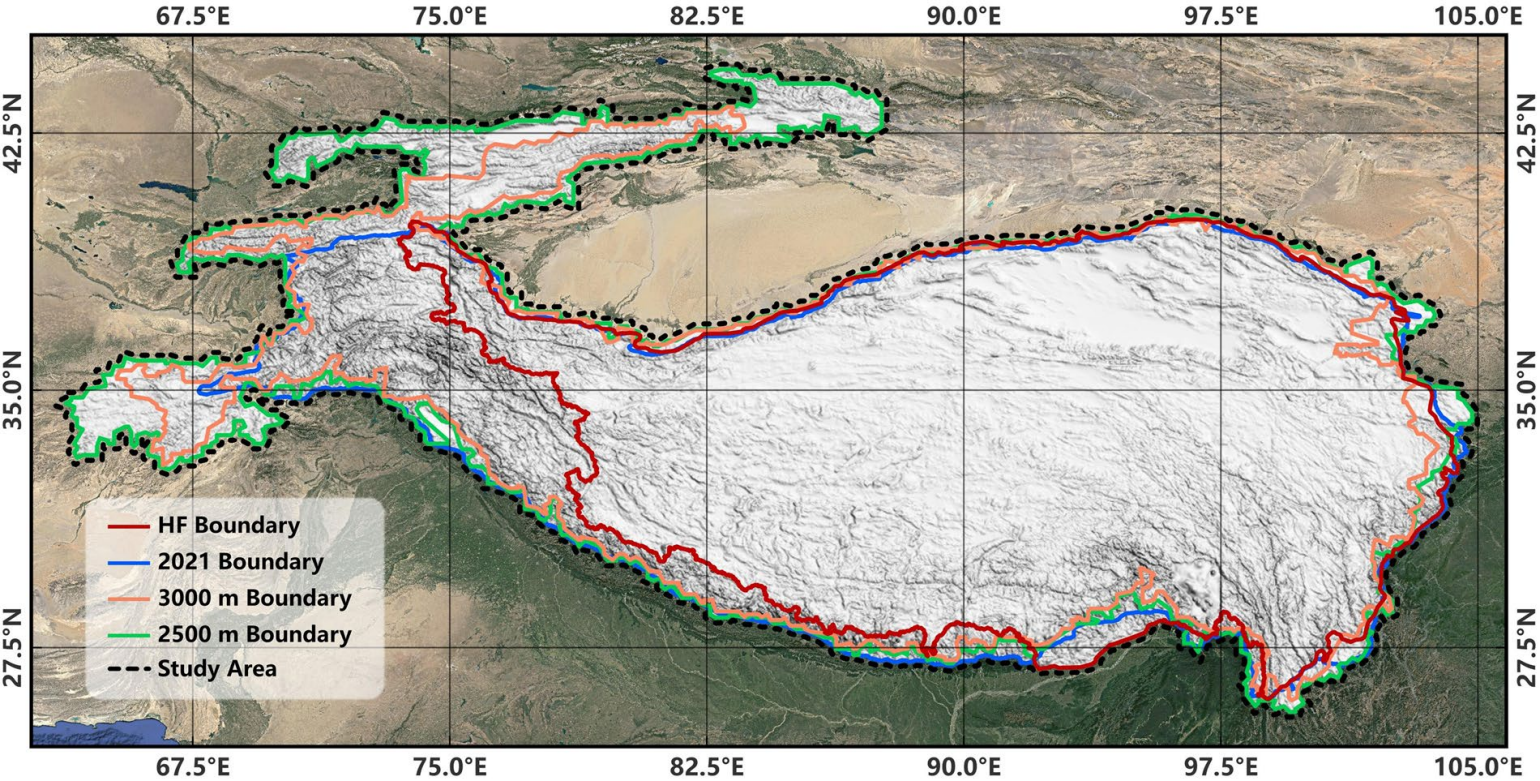
Study Area (Unified QTP Boundary).

Ensuring the equality of data users necessitated a comprehensive approach to define the study area. Therefore, a unified QTP boundary was created by combining the High Frequency (HF) Boundary, the 2021 Boundary, and the 2500 m and 3000 m boundaries^60^ (Fig. 1). Details of these boundaries are given in Supplementary Section 2. This unified boundary encompasses an area of about 3.6 million km^2^, spans latitudes 25.62°N to 44.36°N and longitudes 63.79°E to 104.84°E. This integration approach aims to provide a holistic description of the QTP.

**Fig. 2:**
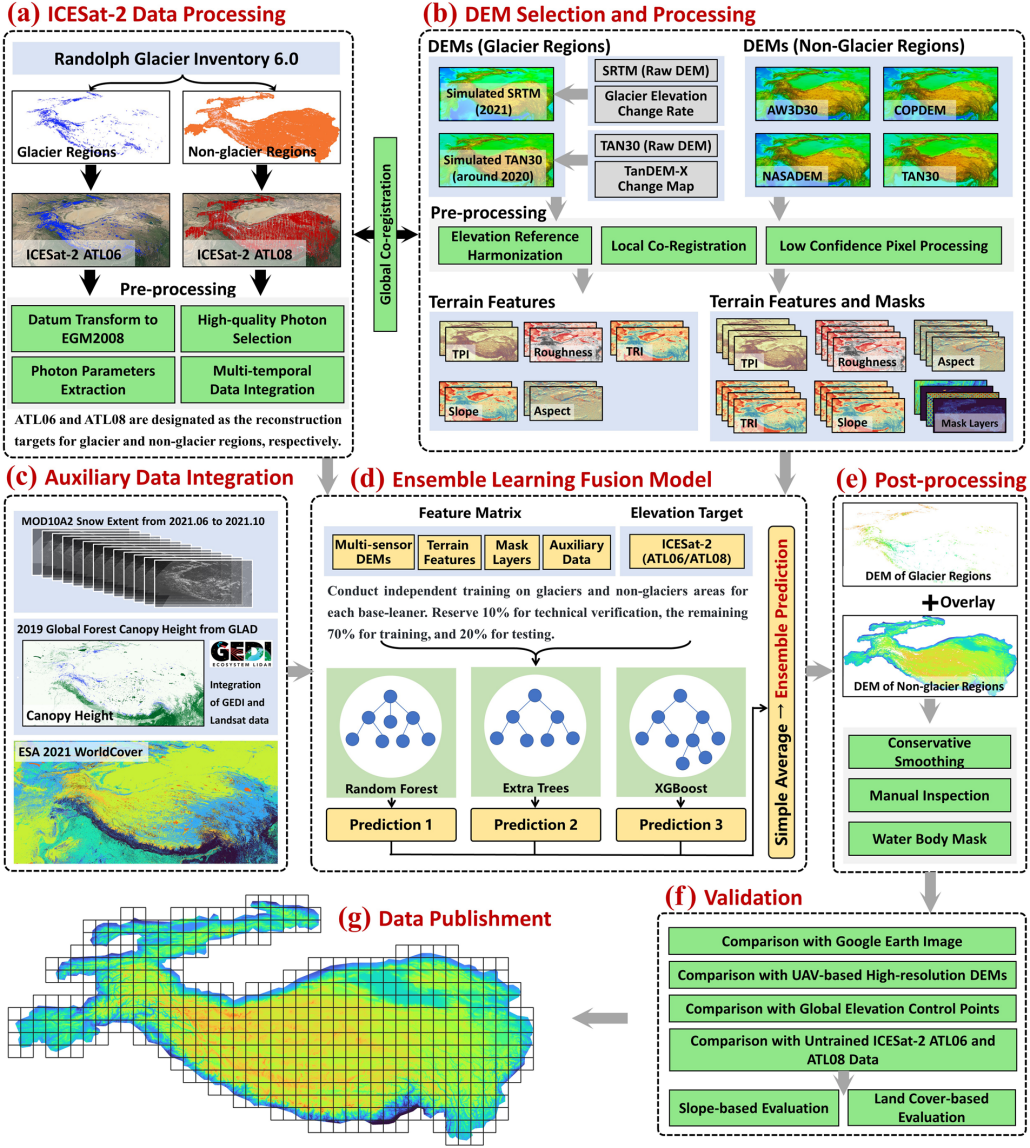
Workflow for HQTP30 Generation.

The construction of HQTP30 consists of a comprehensive workflow that seamlessly integrated LiDAR and multi-sensor DEMs (Fig. 2): (a) ICESat-2 data processing; (b) DEM selection and processing for glacier and non-glacier regions; (c) integration of auxiliary data; (d) construction of ELF model for accurate terrain estimation; (e) post-processing; (f) Evaluation based on multi-sensor data; (g) data publication.

### ICESat-2 data processing

ICESat-2 serves as elevation references in the DEM fusion process. The ICESat-2’s primary instrument, ATLAS, precisely measures the Earth’s surface elevation at 70 cm intervals along the satellite’s track^61^. ATLAS generates approximately 10,000 laser pulses per second, and features six laser beams, organized in 3 pairs, with a 2.5 km spacing in the along-track direction and a 90 m spacing in the across-track direction between beams within each pair. ICESat-2 orbits with a 91-day period, covering latitudes from 88°N to 88°S.

**Fig. 3:**
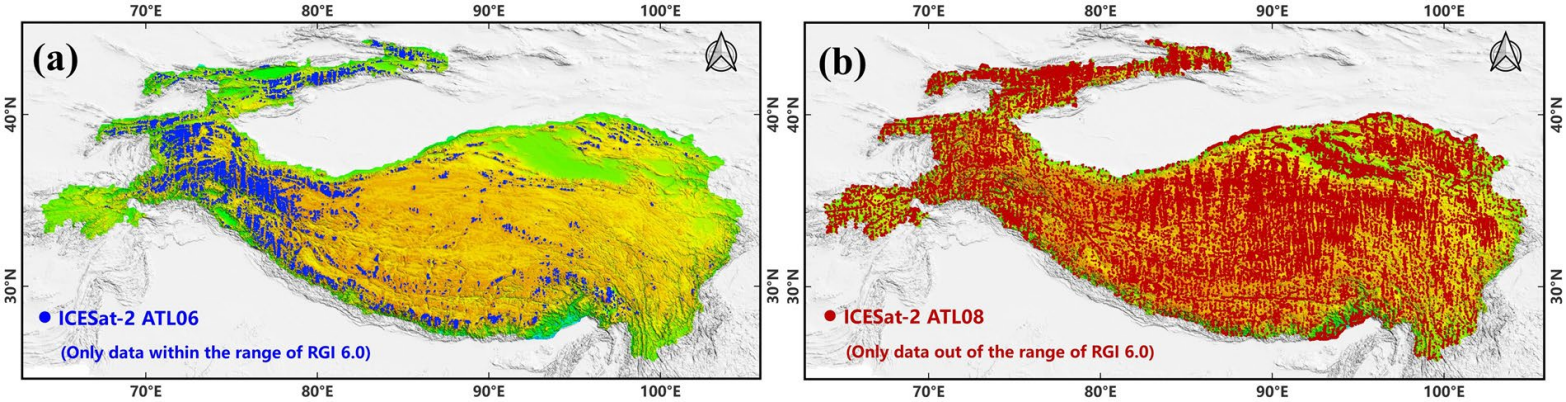
ICESat-2 over QTP: (**a**) ATL06 in Glacier Regions. (**b**) ATL08 in Non-glacier Regions.

The ICESat-2’s ATL03 photon undergoes processing to yield the ATL06 and ATL08 data, which offer more refined elevation data. The ATL06 (Fig. 3a) provides ice sheets or glacier elevation along the satellite’s track, whereas ATL08 (Fig. 3b) offers ground and canopy heights^62^. ATL06 has been widely used in assessing glacier mass balances^63^, ice surface elevation measurement^64^, analysis of ice shelf fractures^56,65,66^, and snow depth retrievals^67,68^. Accuracy assessment of ATL06 asserting its superior precision over any prior elevation measurement products^69^. Meanwhile, ATL08 has been extensively employed for canopy height retrieval^70–72^, tidal flat terrain inversion^53^, and mitigating vegetation impact in DEMs^36^. The terrain heights inverted from ATL08 align with airborne LiDAR datasets, with reported elevation uncertainties ranging between 0.2 m to 0.73 m^49,71,73^. Given the advantages of ATL06 and ATL08 and recognizing the difference in elevation change rates between the glacier and stable non-glacier regions, ATL06 and ATL08 were specifically employed to address elevation correction challenges in these areas. Furthermore, the Randolph Glacier Inventory (RGI) 6.0^74^ was utilized to delineate the boundaries of glacier regions.

#### Photon parameters extraction

The photon parameters were first extracted, and only high-quality photons were selected owing to the inherent noise and uncertainty within laser altimeters^75,76^.

For ATL06, the *latitude*, *longitude*, *atl06_quality_summary*, *delta_time*, *h_li*, and *h_li_sigma* parameters were extracted from the raw granules. The *h_li* represents the glacier surface elevation, while *h_li_sigma* indicates the elevation uncertainty derived from a statistical model based on signal strength and surface slope. Only photons with *h_li_sigma* < 5 m were considered, a criterion that surpasses the COPDEM accuracy validation requirement of 7.5 m^20^. Additionally, high-confidence photons with *atl06_quality_summary* = 0 were reserved. Photon heights deviating more than 200 m from all the DEMs were excluded^54^.

For ATL08, the *latitude*, *longitude*, *h_te_best_fit*, *h_te_uncertainty*, and *h_canopy_height* parameters were extracted from the raw granules. Here, *h_te_best_fit* represents surface elevation, while *h_te_uncertainty* indicates elevation uncertainty. We only selected photons with *h_te_uncertainty* < 5 m. Excluded photons with *h_canopy_height* > 10 m, which suggests potential interference from the canopy. Similarly, photons that differed from the DEMs by more than 200 m were deleted.

Subsequently, elevation was transformed from World Geodetic System 1984 (WGS84) orthometric heights to Earth Gravity Model 2008 (EGM2008) geoid heights:

1 $$\begin{array}{c}{H}_{EGM2008}={H}_{WGS84}-NG{H}_{EGM2008}\end{array}$$

Where *NGH*_*EGM*2008_ represents the geoid undulation between the equipotential surface of EGM2008 and the WGS84 ellipsoid.

The ATL06 and ATL08 were not screened based on the beam energy (strong/weak) or acquisition time (day/night). The ATL08, study reports a Mean Absolute Error (MAE) discrepancy of 0.13 m to 0.15 m between strong and weak beams^77^. Specifically, the MAE discrepancy between day and night for strong beams is 0.04 m, while for weak beams is 0.02 m. The ATL06, no significant difference was observed in the measurement of ice elevation between strong and weak beams, and beam intensity did not systematically affect measurement accuracy^62,78,79^. Generally, the trajectories of strong and weak beams showed high consistency^80^. Considering the inherent error presented in the open-access DEMs, the identified error discrepancies due to beam strength and acquisition time are deemed within an acceptable margin. Typically, the amount of data correlates positively with the generalization ability of machine learning models; therefore, it was decided not to filter the ATL06 and ATL08 based on beam energy and acquisition time.

#### Multi-temporal data integration

**Fig. 4:**
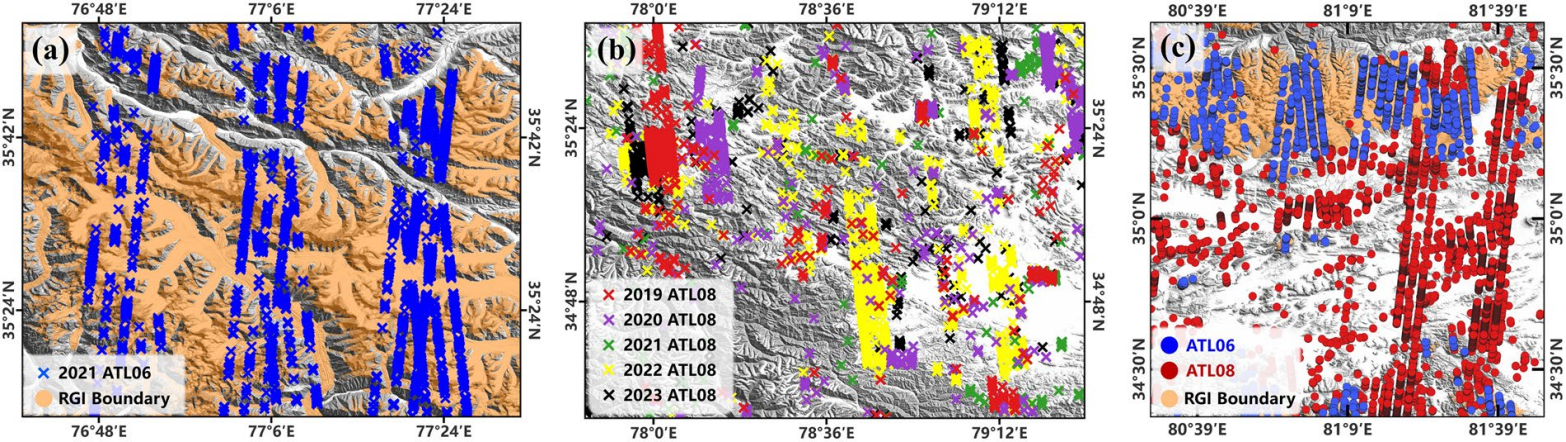
Multi-temporal Data Integration Method: (**a**) ATL06 Data in 2021. (**b**) ATL08 Integration (2019–2023). (**c**) ATL06 and ATL08 Photons in a Specific Region.

Due to the significant disparity in elevation changes observed between glacier and non-glacier regions, different strategies were implemented for the temporal selection of ATL06 and ATL08. It is widely recognized that elevation changes within QTP glacier regions are substantially greater than in non-glacier regions^26^. Therefore, for glacier region, only ATL06 data from June to October 2021 was selected (Fig. 4a). This period was chosen due to the low snow cover from June to October, which mitigates the challenges of elevation measurement caused by snow cover^81^. The choice of limiting the dataset to 2021 aimed to minimize the uncertainty introduced by annual glacial elevation variability.

In contrast, for non-glacier regions, ATL08 data from June to October for each year from 2019 to 2023 was selected (Fig. 4b). The reason for this broader temporal range was to increase the data volume, which is beneficial for the data-driven machine learning model. A potential criticism is that adding data from other years to the 2021 data could disrupt the accurate topographic representation for 2021. In fact, for non-glacier regions of the QTP, the average annual uplift rate is approximately 5 to 6 mm^82–84^, which is insignificant for elevation measurement. Considering the 0.1 m precision of the ATLAS, these changes are essentially undetectable by the laser altimetry.

Furthermore, the integration of multi-temporal data in data-driven machine learning models serves two primary purposes: to increase the photon count, thus reducing the risk of model overfitting, and to expand the coverage of ICESat-2 to include as comprehensive a range of terrain observations as possible across the QTP (Fig. 4c). This strategy yielded a total of 1,588,743 ATL06 photons and 1,191,818 ATL08 photons. Since the typical elevation value (*h_li*) for ATL06 was fitted to the elevation on a 20 m segment, and the typical elevation value (*h_te_best_fit*) for ATL08 was fitted to the elevation on a 100 m segment, the final number of photons acquired is roughly similar even though different temporal screening windows were used.

### DEM selection and processing

SRTM and TAN30 were explicitly selected for the ELF model to reconstruct DEM in glacier regions. AW3D30, COPDEM, TAN30, and NASADEM for non-glacier regions were selected for the ELF model. Elevation reference harmonization, co-registration, low-confidence pixel processing, and terrain feature extraction are performed for all selected DEMs.

#### DEM selection in glacier regions

The primary challenge in selecting glacier region DEMs is the uncertainty of observation time. The SRTM provides a 15-day snapshot of the Earth’s surface in 2000. In contrast, other DEMs observed the QTP with wide temporal coverages: AW3D30 (2006–2011), COPDEM (2010–2015), and NASADEM (initially used 2000 data but was later rectified by ICESat-1 from 2003 to 2009).

Romain *et al*. employed NASA’s 20-year archive of ASTER stereo imagery, modern photogrammetric techniques, and specially developed statistical methods to generate and bias-corrected nearly 500,000 glacier 30 m DEMs^57^. These DEMs then revealed the average elevation change rate across the glaciers of the QTP from 2000 to 2020, independent of any open-access DEMs. Assuming this elevation change rate could be extended to 2021, an approximate simulation of the 2021 terrain is generated using the following formula:

2 $$\begin{array}{c}{{\rm{SRTM}}}_{2021}={\rm{SRTM}}+T\cdot V,\quad V\in \left(-15{\rm{m}}\,{{\rm{yr}}}^{-1},15{\rm{m}}\,{{\rm{yr}}}^{-1}\right)\end{array}$$

SRTM and SRTM_2021_ represent the original SRTM and simulated 2021 SRTM, respectively. *T* denotes the period, which is 21 years for SRTM, and *V* represents the annual glacier change rate. Over the past two decades, the interannual glacier change rate on the QTP has been observed as follows: in the eastern region, −0.317 ± 0.027 m yr^−1^ ^85^; in the central region, 1.12 m yr^−1^ and -0.56 m yr^−1^ ^86,87^, and in the western region, −0.30 ± 0.07 m yr^−1^ ^88^. Furthermore, substantial errors have been identified in areas where the average elevation change rate exceeds ± 15 m yr^−1^, based on error measurement layers provided by Romain *et al*. Consequently, *V* has been conservatively set within the range of (−15m yr^−1^, 15m yr^−1^) to preserve the most reliable signals, which represent approximately 99.9998% of the data, effectively compressing some of the most extreme estimates. The penetration effect of SAR sensors is not considered here, as it is hard to determine the height of SAR penetration, and ELF models can correct minor errors introduced by penetration.

In December 2023, the German Aerospace Center (DLR) released the TanDEM-X 30 m Edited DEM (TAN30) and the TanDEM-X 30 m DEM Change Map (DCM). TAN30 was generated based on TanDEM-X observation data collected between 2010 and 2015, utilizing the latest data processing techniques. The TAN30 DCM includes differences between new elevation observations in glacier regions from 2016 to 2022 (in QTP, mostly 2020) and the TAN30.

To update TAN30 with the latest elevation observation data, the layer marked “LAST” from the TAN30 DCM was used. Additionally, the Height Accuracy Indication (HAI) layer provided with the DCM was employed to identify unreliable areas. Elevation changes with errors exceeding 1.5 m, as indicated by HAI, were excluded. This 1.5 m threshold was determined through visual assessment.

Given that TAN30’s earliest observation was in 2010 and the TAN30 DCM’s latest observation was in 2022, the maximum period of elevation change is 12 years. An annual change threshold of ±15 m yr^-1^, similar to that of the previously mentioned SRTM, was applied. Therefore, changes exceeding ±180 m (12 × ±15 m yr^−1^) in the DCM were compressed to within ± 180 m. Finally, the updated elevation model, TAN30_*update*_, was generated by adding the TAN30 and DCM data:

3 $$\begin{array}{c}{\rm{TAN}}3{0}_{update}={\rm{TAN}}30+{\rm{DCM}},\quad {\rm{DCM}}\in \left(-180{\rm{m}},180{\rm{m}}\right)\end{array}$$

The SRTM_2021_ and TAN30_*update*_ serve as DEMs for the fusion DEMs in glacier regions.

#### DEM selection in non-glacier regions

For non-glacier regions, the reliability of a DEM is primarily reflected in its ability to represent terrain. Over time, new data have been used to process and fill gaps in the original data or to update DEMs in relative rapidly changing regions. This means that the main differences between DEMs lie in quality rather than observation time. Thus, the selection of DEMs for non-glacier regions can be primarily based on quality, allowing for the fusion of the highest quality DEMs without undue concern for temporal variations. Therefore, several DEMs (including ASTER GDEM, AW3D30, COPDEM, TAN30, NASADEM, SRTM, and Multi-Error-Removed Improved-Terrain DEM (MERIT DEM)^11–13,16,89,90^ were selected for pre-validation for further consideration.

Pre-validation aimed to select DEMs based on verified data amidst ongoing debates about their relative accuracies. The HAGECPD^91^ (derived from ICESat-1)^92^ and ICESat-2 ATL08 were selected as benchmarks. No regional high-resolution DEMs were utilized due to the inability of a small sample size to represent the complex landscape of the QTP (Supplementary Section 1). The results (Supplementary Section 3, 4) indicate that four DEMs show accuracy advantages in non-glacier regions, which are selected: AW3D30, COPDEM, TAN30, and NASADEM. Among these, AW3D30 relies on optical stereo photogrammetry technology, whereas COPDEM, TAN30, and NASADEM are derived from SAR technology. All selected DEMs have a spatial resolution of 1 arc-second (≈ 30 m).

The selected DEMs incorporate mask layers for quality control. The “Stack Number File” (STK) of AW3D30 refers to the count of scenes for generating AW3D30. The Edit Data Mask (EDM) of COPDEM describes the processing undertaken for each pixel. The Number (NUM) layer of NASADEM indicates the data source for each pixel. The Height Error Map (HEM) of TAN30 serves as a potential error range layer. The AW3D30 STK, COPDEM EDM, NASADEM NUM, and TAN30 HEM were integrated into the ELF model (detailed later).

#### Elevation reference harmonization

The AW3D30, COPDEM, TAN30, NASADEM, and SRTM refer to the WGS84 spatial coordinate system, while utilizing different elevation references. Specifically, COPDEM and TAN30 utilize the EGM2008 geoid, while the AW3D30, NASADEM, and SRTM employ the Earth Gravity Model 1996 (EGM96) geoid. The SRTM, AW3D30, and NASADEM were converted to EGM2008 orthometric heights (detailed in Supplementary Section 5), considering its status as the most recent and precise Earth gravity model^93,94^.

#### Co-registration

**Fig. 5:**
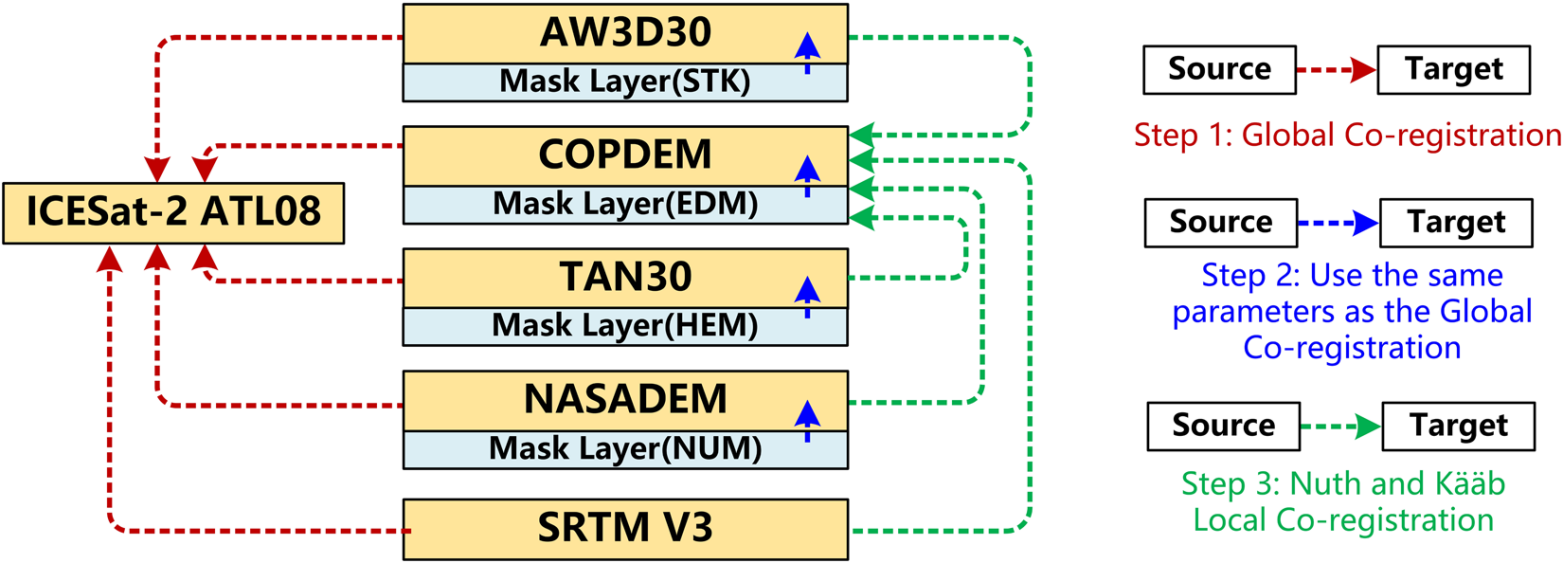
Spatial Co-registration of ICESat-2 with DEMs and Mask Layers.

Achieving precise spatial alignment between DEMs and ICESat-2 is of utmost importance. A co-registration workflow for data alignment is presented (Fig. 5).

Global Co-registration: Each DEM is globally registered to the ATL08 data. The steps are: (a) Overlay the ATL08 photons with DEM, using the Bilinear interpolation method to sample the values of ATL08 photons at corresponding positions in the DEM. (b) Calculate the MAE between the DEM values and the ATL08 elevation. (c) Shift the DEM data in steps of 0.5 m within a spatial range of 50 m × 50 m, and repeat steps (a) and (b). (d) Determine the optimal spatial correction position for the DEM relative to ICESat-2 based on the minimum MAE. The offset parameters determined for each DEM were applied to the corresponding mask layers.

Local Co-registration: Despite effective global co-registration, it does not address non-systematic errors from DEM noise, artifacts, or data collection inconsistencies. To refine DEM alignment at a finer scale and improve terrain feature extraction, we apply local co-registration using the Nuth and Kääb algorithm^95^. This procedure aligns AW3D30, TAN30, NASADEM, and SRTM with COPDEM based on COPDEM’s superior geolocation accuracy (officially reported within 5 m)^96^. Testing experiments show that discrepancies in land features can introduce artifacts and noise in the DEM fusion results, underscoring the importance of this procedure. The maximum offset set in the Nuth and Kääb algorithm is 50 m, consistent with global co-registration.

#### Low confidence pixel processing

AW3D30, COPDEM, and NASADEM employ interpolation methods to fill void areas, but the accuracy of these interpolated values remains uncertain. To tackle this issue, the AW3D30’s Format definition change of mask (MSK) file, COPDEM’s Editing Mask (EDM) file, and TAN30’s Editing Mask (EDM) file were employed to detect interpolated regions. Interpolated or void regions in each DEM with length and width exceeding 10 pixels were filtered out. If available, the values from the other DEMs (mean value if multiple) were used to fill the filtered regions. If no DEM could provide actual observations for a particular region, the original interpolated value was retained, as no sensor observations exist. It is worth noting that if only one actual observation exists in a region, all values in other DEMs are replaced by it. This approach is favored over retaining the interpolated values since actual observations generally offer greater reliability.

#### Terrain feature extraction

Disparities in multi-sensor DEMs do not solely stem from data quality but are significantly influenced by the intricate and diverse nature of the terrain features^97^. To enhance the accuracy of error estimation and fusion results, a set of variables representing terrain features has been carefully chosen as input parameters for the ELF model, including Slope, Aspect, Roughness, Terrain Ruggedness Index (TRI)^98^, and the Nine-Neighborhood Average (NNA) value (derived by averaging the values of the current pixel and its surrounding pixels). Slope and Aspect represent fundamental terrain attributes, while Roughness and TRI provide insights into terrain complexity and micro-variations. Incorporating NNA value into the ELF model aims to consider the correlation between the current pixel and its neighboring pixels, assisting the model in combating the inherent deformation in the DEMs.

Incorporating these terrain parameters is rooted in the scientific hypothesis that variations in DEM from different sources may be linked to terrain characteristics. Consequently, including these features enables the ELF model to more effectively align error distributions and patterns, thereby enhancing the accuracy of fusion outcomes.

### Auxiliary data integration

To comprehensively analyze the intricate terrain of the QTP, auxiliary data was integrated into the ELF model (Fig. 2c), including the European Space Agency (ESA) 2021 WorldCover^99,100^, the MODIS/Terra Snow Cover 8-Day L3 Global 500 m Grid (MOD10A2)^101^ spanning from June to October 2021 (comprising 13 images), and the 2019 Global Forest Canopy Height Map (GFCHM)^102^ derived from Global Ecosystem Dynamics Investigation (GEDI) and Landsat. This comprehensive approach accounts for the impacts of snow accumulation, land cover types (e.g., Forests), and canopy height on the accuracy of DEM. A detailed description of all data utilized in the ELF model is provided in Table 1.

**Table 1:** Data Details for ELF Model.

Item	Version	Main Survey Time	References	Resolution	Sensor Type	Data Type
AW3D30	V3.2	2006–2011	WGS84/EGM96	30 m	Stereo Optical	16-bit int
COPDEM	V2.1	2010–2015	WGS84/EGM2008	30 m	SAR X-band	32-bit float
TAN30	2023	2010–2015	WGS84/ EGM2008	30 m	SAR X-band	32-bit float
NASADEM	V1.1	2000	WGS84/EGM96	30 m	SAR C-band	16-bit int
SRTM	V3	2000	WGS84/EGM96	30 m	SAR C-band	16-bit int
ICESat-2 ATL06	V006	2018 - Now	WGS84/Ellipsoid	17 m	Laser Altimeter	32-bit float
ICESat-2 ATL08	V006	2018 - Now	WGS84/Ellipsoid	17 m	Laser Altimeter	32-bit float
WorldCover	V200	2020–2021	WGS84	10 m	SAR C-band; Multi-spectral	8-bit int
GFCHM	2021	2019	WGS84	30 m	Laser Altimeter; Multi-spectral	8-bit int
MOD10A2	V61	2000 - Now	WGS84	500 m	Multi-spectral	8-bit int

The MOD10A2 has been resampled to 30 m using the nearest neighbor method. Similarly, the WorldCover has undergone majority resampling to 30 m. The MOD10A2 product provides the maximum extent of snow cover observed within eight days. One-hot encoding classifies the MOD10A2 into two distinct categories: snow-covered and snow-free. For glacier regions characterized by homogeneous land cover and an absence of forest structures, only encoded MOD10A2 was utilized as auxiliary information in the ELF model. In this scenario, WorldCover and GFCHM were intentionally excluded from consideration as they are irrelevant to glacier regions.

### Ensemble learning fusion (ELF) model

ELF model is designed for terrain estimation in glacier and non-glacier regions. The model firstly constructs two feature matrices representing complex physical terrain characteristics by merging DEMs, terrain features, mask layers, and other datasets. The ELF model employs three tree-based regression methods as base learners. Each of these learners all undergo independent training in glacier and non-glacier regions to account for discrepancies between recent LiDAR observations and open-access DEMs. Training establishes a nonlinear mapping between high-accuracy elevation values and the constructed feature matrices. During prediction, the model uses these mappings to simulate the elevation of each pixel. A simple averaging approach combines predictions from the base learners, enhancing the model’s robustness.

Specifically, let ${{\boldsymbol{D}}}_{glc}\in {{\mathbb{R}}}^{{m}_{1}\times H\times W}$ represent the DEMs for glacier regions (SRTM_2021_, TAN30_*update*_), *H* and *W* denote the height and width of DEM, *m*_1_ is the number of selected glacier DEMs, here ${m}_{1}=2$. Let ${{\boldsymbol{D}}}_{stb}\in {{\mathbb{R}}}^{{m}_{2}\times H\times W}$ represent the selected DEMs (AW3D30, COPDEM, TAN30, NASADEM) for stable non-glacier regions, where *m*_2_ is the number of DEMs, here *m*_2_=4. Let ${{\boldsymbol{T}}}_{glc}\in {{\mathbb{R}}}^{5{m}_{1}\times H\times W}$ be the terrain features extracted from ***D***_*glc*_. Let ${{\boldsymbol{T}}}_{stb}\in {{\mathbb{R}}}^{5{m}_{2}\times H\times W}$ be the terrain features extracted from ${{\boldsymbol{D}}}_{stb}$. Additionally, ${{\boldsymbol{M}}}_{stb}\in {{\mathbb{R}}}^{{m}_{2}\times H\times W}$ represents the Mask layers of ***D***_*stb*_. ${{\boldsymbol{A}}}_{G}\in {{\mathbb{R}}}^{1\times H\times W}$ as the GFCHM, and ${{\boldsymbol{A}}}_{W}\in {{\mathbb{R}}}^{1\times H\times W}$ as the WorldCover. Furthermore, let ${{\boldsymbol{A}}}_{S}\in {{\mathbb{R}}}^{n\times H\times W}$ represent the MOD10A2, where *n* denotes the number of multi-temporal image layers.

Subsequently, two feature matrices ${{\bf{X}}}_{1}\in {{\mathbb{R}}}^{\left(6{m}_{1}+n\right)\times H\times W}$ and ${{\bf{X}}}_{2}\in {{\mathbb{R}}}^{\left(7{m}_{2}+n+2\right)\times H\times W}$ are constructed as follows:

4 $$\begin{array}{c}{{\bf{X}}}_{1}={{\boldsymbol{D}}}_{glc}\oplus {{\boldsymbol{T}}}_{glc}\oplus {{\boldsymbol{A}}}_{S}\end{array}$$

5 $$\begin{array}{c}{{\bf{X}}}_{2}={{\boldsymbol{D}}}_{stb}\oplus {{\boldsymbol{T}}}_{stb}\oplus {{\boldsymbol{M}}}_{stb}\oplus {{\boldsymbol{A}}}_{G}\oplus {{\boldsymbol{A}}}_{W}\oplus {{\boldsymbol{A}}}_{S}\end{array}$$

Here, $\oplus$ denotes the matrix concatenation operation. It is evident that for ***X***_1_ applicable to glacier areas, only Snow Cover (***A***_*S*_) serves as auxiliary data. Forest Height (***A***_*G*_) and WorldCover (***A***_***W***_) are irrelevant to glacier regions, theoretically assuming uniform values. Any outliers, if present, could likely be attributed to data errors.

For ATL06 and ATL08, 10% of the data is randomly reserved for technical validation. The remaining 70% is used for the training and 20% for the testing sets. Terrain elevation from ATL06 and ATL08 (i.e., *h_li* and *h_te_best_fit*) is utilized as target values for ELF models in glacier and non-glacier regions, denoted as ${{\bf{Y}}}_{glc}\in {{\mathbb{R}}}^{p}$ and ${{\bf{Y}}}_{stb}\in {{\mathbb{R}}}^{q}$, respectively. *p* and *q* are the number of photons. Define ***X***_*glc*_ as the sampling of **Y**_*glc*_ at spatial positions corresponding to **X**_1_, and define **X**_*stb*_ as the sampling of **Y**_*stb*_ at spatial positions corresponding to **X**_2_. Bilinear sampling is used for ***D***_*glc*_, ***D***_*stb*_ ***T***_*glc*_, ***T***_*stb*_, and ***A***_*G*_, while Nearest Neighbor sampling is used for other layers.

Subsequently, three tree-based regression methods serve as base learners: Random Forest^103^, ExtraTrees^104^, and XGBoost^105^, denoted as *f*_RF_, *f*_ET_, *f*_XGB_. Due to significant differences in terrain features and elevation distribution between the glacier and non-glacier regions, training and prediction in these two regions are performed independently. By fitting the training data, these base learners establish nonlinear mapping relationships between **Y**_ATL06_ (or **Y**_ATL08_) and **X**_ATL06_ (or **X**_ATL08_). The base learner’s parameters are optimal values obtained by grid search. During the prediction phase, these three base learners utilize the learned nonlinear mapping relationships to perform per-pixel elevation simulations in the glacier (or non-glacier) regions and use a simple averaging method to combine the predictions, as follows:

6 $$\begin{array}{c}{\widehat{{\bf{Y}}}}_{glc}={g}_{average}\left({f}_{{\rm{RF}}}\left({{\bf{X}}}_{{\rm{ATL}}06}\right),{f}_{{\rm{RF}}}\left({{\bf{X}}}_{{\rm{ATL}}06}\right),{f}_{{\rm{XGB}}}\left({{\bf{X}}}_{{\rm{ATL}}06}\right)\right)\end{array}$$

7 $$\begin{array}{c}{\widehat{{\bf{Y}}}}_{stb}={g}_{average}\left({f}_{{\rm{RF}}}\left({{\bf{X}}}_{{\rm{ATL}}08}\right),{f}_{{\rm{RF}}}\left({{\bf{X}}}_{{\rm{ATL}}08}\right),{f}_{{\rm{XGB}}}\left({{\bf{X}}}_{{\rm{ATL}}08}\right)\right)\end{array}$$

Where *g*_*average*_ represents the average method used to integrate the predictions of various base learners. Subsequently, the data for glacier and non-glacier regions are combined spatially:

8 $$\begin{array}{c}\widehat{{\bf{Y}}}=\left\{\begin{array}{l}{\widehat{{\bf{Y}}}}_{glc},if\;data\;is\;in\;glacier\;region\\ {\widehat{{\bf{Y}}}}_{stb},if\;data\;is\;in\;non \mbox{-} glacier\;region\end{array}\right.\end{array}$$

Integrating multiple learners helps reduce the risk of overfitting and enhances the system’s robustness, which improves the overall predictive performance by combining the opinions of multiple regression algorithms. For interpretation of features in the model, please refer to Supplementary Section 6.

### Smoothing and permanent water body processing

**Fig. 6:**
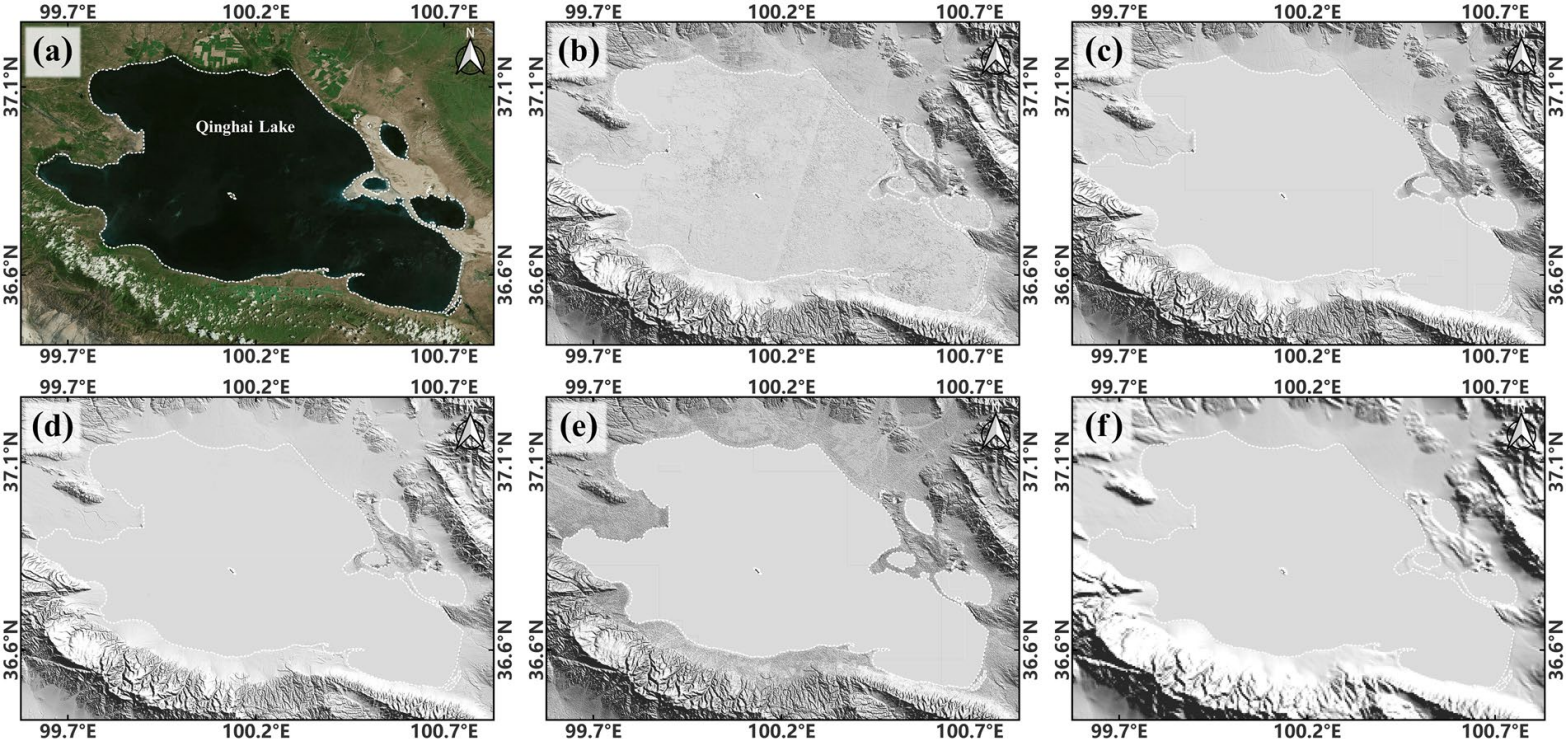
Terrain Representation in Qinghai Lake using Open-access DEMs: (**a**) Optical Image Reference. (**b**) AW3D30. (**c**) COPDEM. (**d**) TAN30. (**e**) NASADEM. (**f**) ETOPO2022.

A Conservative Smoothing filter^106^ was globally utilized using the Whitebox software^107^ to eliminate potential outliers. Subsequently, a manual inspection of the HQTP30 was performed to ensure the absence of significant errors in the DEM. Lakes cover approximately 1.18% of the QTP surface, while the currently available DEMs do not offer sufficient information for underwater terrain modeling (Fig. 6). While ICESat-2 ATL13^108^ is specifically designed for water modeling, it provides solely for water surface elevations, lacking water depth information. Faced with these constraints, we tried integrating Earth Topography 2022 (ETOPO2022)^109^, which claims to provide global bedrock elevations. Nonetheless, assessments revealed the inadequacy of pertinent bedrock elevation for the QTP within ETOPO2022 (Fig. 6f).

The extent of lakes on the QTP has undergone noticeable changes^110,111^. Therefore, the HydroLAKES^112^ and Global Surface Water (GSW)^113^ were combined to define the 2021 QTP lake boundaries. HydroLAKES provided initial lake locations, while the GSW seasonality layer helped refine these boundaries. Initially, lakes were located using the HydroLAKES. The permanent waterbodies from the GSW seasonality layer were then extracted and vectorized. Then, the HydroLAKES will be overlayed with the permanent waterbodies, retaining only the permanent waterbodies that overlapped with HydroLAKES, which will serve as the new lake boundaries for 2021. A manual inspection using Google Earth Map was performed to eliminate potential errors. Subsequently, only lakes covering more than 100 pixels were retained. The DEM pixels within these lakes were smoothed using the mean value of all bilinear elevations at the intersection points of the lake boundaries with HQTP30. Masking was not performed for newly formed QTP lakes to preserve potential underwater terrain detected in early terrain observations.

## Data Records

The dataset is available for free download at figshare^114^. It represents a high-accuracy terrain model of the QTP within the WGS84 coordinate system (EPSG: 4326) and is referenced to the EGM2008 Geoid. This dataset encompasses the entire QTP area with a spatial resolution of 1 arc-second (≈30 m) and is divided into 494 tiled files. Each tiled file measures 1° by 1° and is stored in Geotiff format. The naming convention for these files follows a structured format: HQTP30_Naa_Ebbb.tif, where “aa” corresponds to the latitude, and “bbb” corresponds to the longitude. For example, HQTP30_N30_E090 represents a tiled file covering the region from N30° to 31° and E90° to 91°. To enhance user accessibility and facilitate a quick assessment of the dataset’s quality and characteristics, 3D rendering previews are provided for each tiled file.

## Technical Validation

We conducted a thorough validation using four distinct datasets to assess the elevation accuracy and terrain detail performance of HQTP30. Dataset a comprises a high-resolution orthophoto from Google Earth Map for visual comparison. Dataset b consists of three high-resolution regional DEMs obtained from UAV surveys. Dataset c includes high-quality control points derived from ICESat-1, while dataset d incorporates ICESat-2 ATL06 and ATL08 photons excluded from the ELF model. Despite GEDI and ICESat-2 being spaceborne LiDAR data, we opted against using GEDI for validation. Prior research indicates that GEDI, despite its advantage in canopy height measurements, exhibits higher surface elevation errors compared to ICESat-2^77,115^.

The primary function of dataset a is to facilitate visual comparisons in areas lacking reference. Therefore, for the AW3D30, COPDEM, NASADEM, and TAN30, we selected 20 typical rugged areas and compared them visually with HQTP30. However, this intuitive comparison clearly lacks a quantitative representation of elevation, and these quantitative comparisons were made in datasets b, c, and d. For the validation using dataset b, we resampled the UAV-based DEMs to 1 arc-second through pixel averaging to meet the statistical requirements of the DEMs to be validated. Subsequently, pixel-level accuracy validation was executed. Regarding the accuracy validation using datasets c and d, we utilized bilinear interpolation to extract elevation values from the DEMs of the four pixels closest to the center of the photon footprint. All validation data were harmonized with the WGS84 coordinate system and the EGM2008 geoid.

### Visual Comparison

**Fig. 7:**
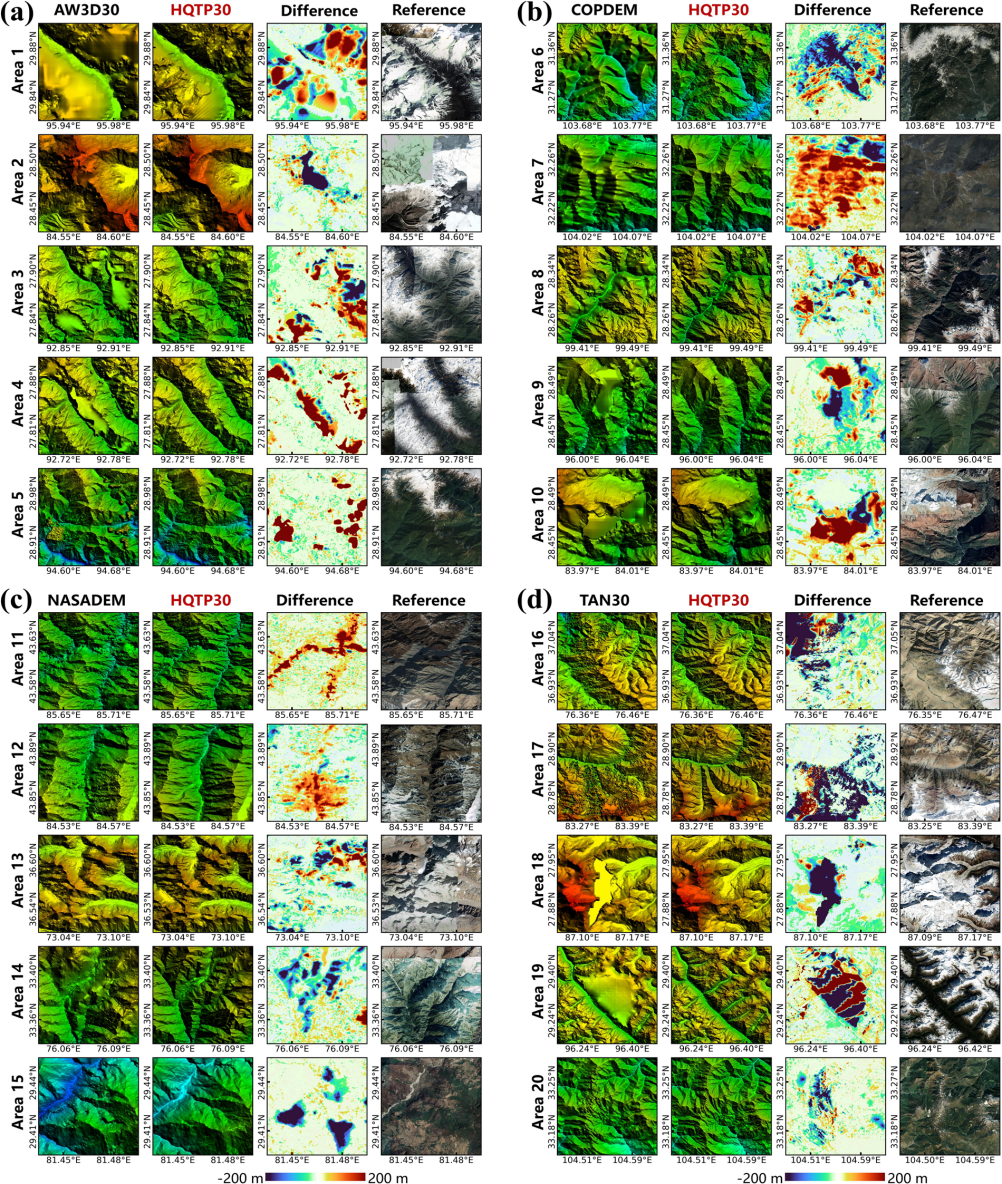
Comparison of HQTP30 and Open-access DEMs in 20 Extremely Rugged Areas with Google Earth Map Orthophoto as Reference. The difference between HQTP30 and existing products is also provided for identifying distinctions. (**a**) Comparison of AW3D30 and HQTP30; (**b**) Comparison of COPDEM and HQTP30; (**c**) Comparison of NASADEM and HQTP30; (**d**) Comparison of TAN30 and HQTP30.

The visual comparison of HQTP30 with other DEMs in 20 complex regions is presented (Fig. 7). Overall, AW3D30 exhibits significant errors in snow-covered regions (Areas 1, 3, 4), possibly due to image-matching issues caused by monotonous ice and snow textures. Additionally, there are some noticeable outliers (Area 5). In contrast, HQTP30 lacks these terrain artifacts, as the ensemble learning model can eliminate terrain anomalies from a single sensor. COPDEM shows striping artifacts in certain areas (Area 7), excessive smoothing in rugged terrain (Area 6, 8), or terrain gaps (Area 9, 10). This phenomenon may be attributed to the inability of side-view images to capture stereo information in those locations. Conversely, HQTP30 does not exhibit these issues; the ensemble learning model can infer or interpolate more realistic terrain information from other available observations.

The primary issue with NASADEM arises from significant noise from the SRTM observation36, a problem avoided in HQTP30. TAN30 provides relatively accurate values in observed regions, but there are extensive noises (Area 16, 17, 20) and even over-smoothed areas (Area 18.19), while HQTP30 eliminates these noises.

### Validation using UAV-derived DEMs

In the rigorous survey within the QTP, efforts are made to seek high-accuracy DEMs that are closely integrated with 2021, have higher accuracy, and cover a wide range of areas. Adhering to these stringent criteria, three regional DEMs were identified, all acquired through UAV-derived Structure from Motion (SfM)^116^ techniques (Table 2).

**Table 2:** Details of UAV-derived DEMs.

No.	Dataset Name	Survey Date	Area	Resolution
1^117^	High Resolution Topography of the Lagtan Anticline	October, 2020	13.25 km^2^	0.06 m
2^118^	Survey of fault south of Song Kul, Kyrgyzstan, 2021	August, 2021	3.41 km^2^	0.12 m
3^119^	Orthophoto and DSM products obtained from UAV aerial survey for the typical talus landform in the Zheduoshan Mountain, China (2020)	August 2020	22.6 km^2^	0.10 m

Quantitative evaluation of open-access DEMs and the HQTP30 across the Lagtan Anticline, the Fault South of Song Kul, and the Zheduoshan Mountain is presented in Table 3. DEMs are evaluated using the MAE and Root Mean Square Error (RMSE) metrics. In the Lagtan Anticline, HQTP30 exhibited a notably reduced error, with an MAE of 1.07 m and an RMSE of 1.35 m. Compared to the state-of-the-art (SOTA) COPDEM, HQTP30 demonstrated reductions of 0.14 m in MAE and 0.25 m in RMSE. Other open-access DEMs exhibited more considerable error ranges, with MAE ranging from 1.21 m to 6.17 m, and RMSE values ranging from 1.60 m to 8.31 m, respectively. In the Fault South of Song Kul, HQTP30 displayed RMSE reductions of 0.27 m compared to the SOTA COPDEM. For the Zheduoshan Mountain, the lowest MAE among open-access DEMs was 3.68 m from COPDEM, and HQTP30 demonstrated a decrease of 0.52 m. The lowest RMSE among open-access DEMs was 4.98 m for AW3D30, while HQTP30 achieved 4.26 m, representing a decrease of 0.72 m compared to AW3D30. Quantitative validation results using UAV-derived DEMs affirm the high accuracy of HQTP30 across a diverse range of topographic scenarios.

**Table 3:** Quantitative Assessment of Open-access DEMs and HQTP30 Using UAV-Derived DEMs.

Survey Area	Metric (m)	ASTER GDEM	AW3D30	COPDEM	TAN30	NASADEM	SRTM	MERIT	HQTP30
Lagtan Anticline	MAE	6.17	2.06	1.21	1.46	2.78	3.07	4.36	**1.07**
	RMSE	8.31	2.70	1.60	1.83	3.66	4.05	5.73	**1.35**
Fault South of Song Kul	MAE	3.32	1.94	**1.13**	1.27	2.92	5.97	3.20	1.32
	RMSE	4.66	2.76	1.86	2.00	3.82	6.87	4.24	**1.59**
Zheduoshan Mountain	MAE	9.42	3.76	3.68	3.72	5.41	4.86	5.10	**3.16**
	RMSE	11.64	4.98	5.16	5.31	7.61	6.64	7.21	**4.26**

**Fig. 8:**
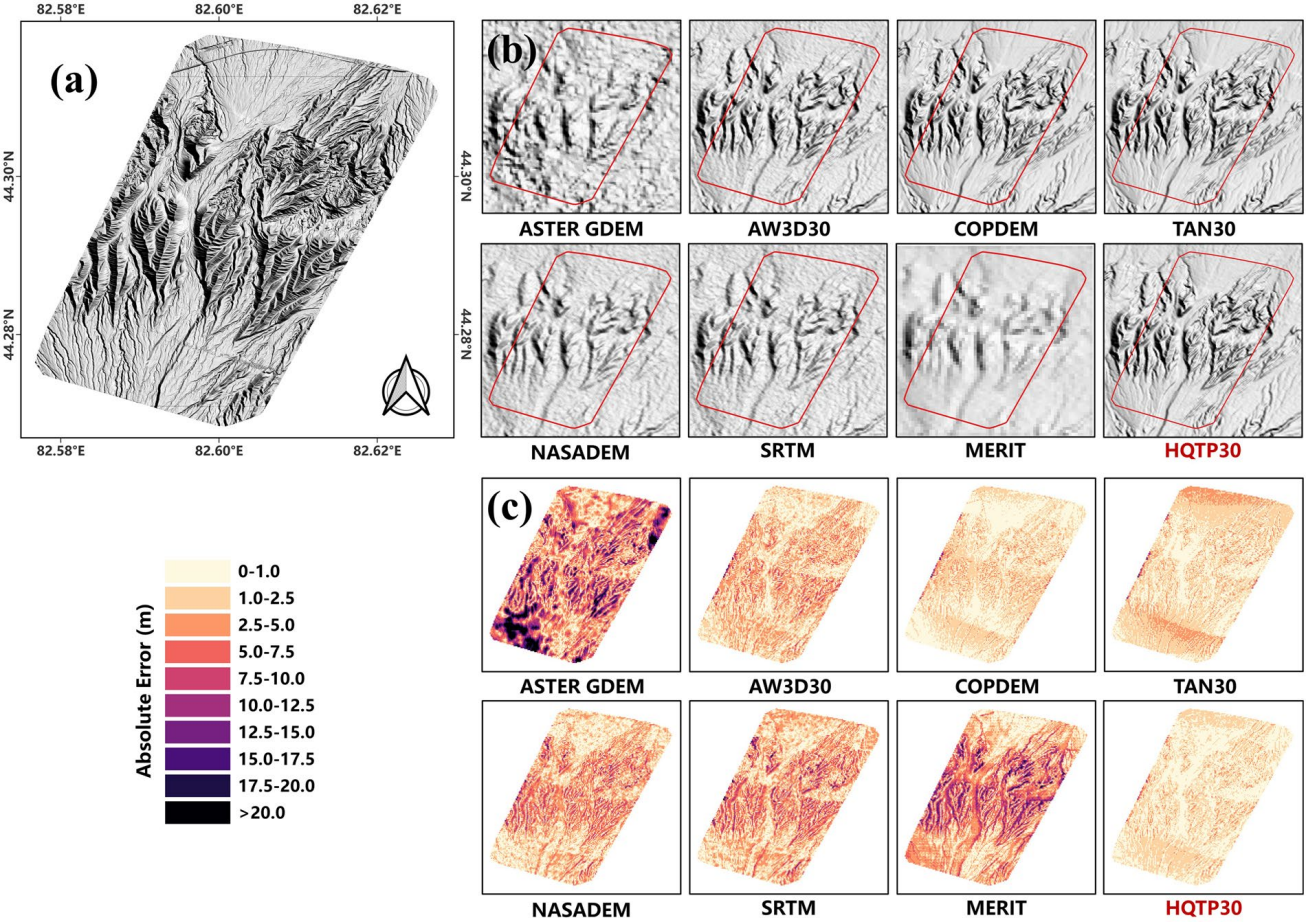
Comparison of UAV-derived DEM, HQTP30, and open-access DEMs in Lagtan Anticline. (**a**) UAV-derived DEM; (**b**) Terrain Rendering Comparison; (**c**) Error Details.

Figure 8 illustrates the 3-dimensional (3D) renderings of the Lagtan Anticline using various DEMs. HQTP30 offers a sharper representation of the topographic features within the Lagtan Anticline (Fig. 8b,c). AW3D30, COPDEM, and TAN30 demonstrate strong topographic characterization abilities, while ASTER GDEM, NASADEM, and SRTM V3 exhibit various potential noise artifacts. Due to the resolution limitations, MERIT could not capture intricate terrain details. Error details show that HQTP30 is less affected by highly rugged terrains, with a lower proportion of pixels displaying significant elevation anomalies. Supplementary Section 7 provides 3D renderings and Error details for the Fault of Song Kul and Zheduoshan Mountain, respectively.

### Validation using control points

The HAGECPD^91^ was employed to quantify the errors of each DEM. HAGECPD exclusively encompasses control points within land areas with slopes less than 25°, excluding snow/ice-covered regions. As a result, it inherently yields a biased estimation. Nevertheless, it offers valuable insights for evaluating DEM accuracy in QTP.

**Fig. 9:**
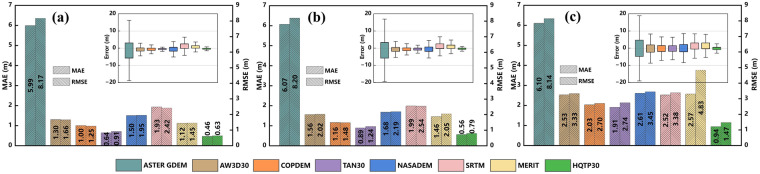
Accuracy assessment using HAGECPD in Different Terrains: (**a**) Flat Terrain (Slope ≤ 2°); (**b**) Hilly Terrain (2° < Slope ≤ 6°); (**c**) Mountainous Terrain (6° < Slope ≤ 25°).

Following a pre-defined terrain classification of HAGECPD, the control points were classified into three categories: flat, hilly, and mountainous (Fig. 9a–c). Subsequently, error metrics were computed for each terrain category. The HQTP30 consistently exhibits the lowest error across all categories (Fig. 9). The ASTER GDEM demonstrates the highest error across various categories. All open-access DEMs display a gradual increase in error as terrain complexity escalates. However, TAN30, AW3D30, and COPDEM exhibit commendable performance across all categories. In comparison, NASADEM and SRTM present a modest performance across all categories, devoid of any conspicuous competitive advantage. Notably, the MERIT, derived from the fusion of SRTM and ALOS observations, experiences a noticeable decrease in accuracy in steep-slope regions.

In flat terrain, HQTP30 exhibits an MAE of 0.46 m and an RMSE of 0.63 m, representing reductions of 28.13% and 30.77%, respectively, compared to the SOTA TAN30. In hilly terrain, HQTP30 displays an MAE of 0.56 m and an RMSE of 0.79 m, representing a 37.08% and 36.29% reduction compared to TAN30. In mountainous terrain, HQTP30 showcases an MAE of 0.94 m and an RMSE of 1.47 m, which is 50.79% and 46.35% lower than TAN30.

### Validation using ICESat-2

**Fig. 10:**
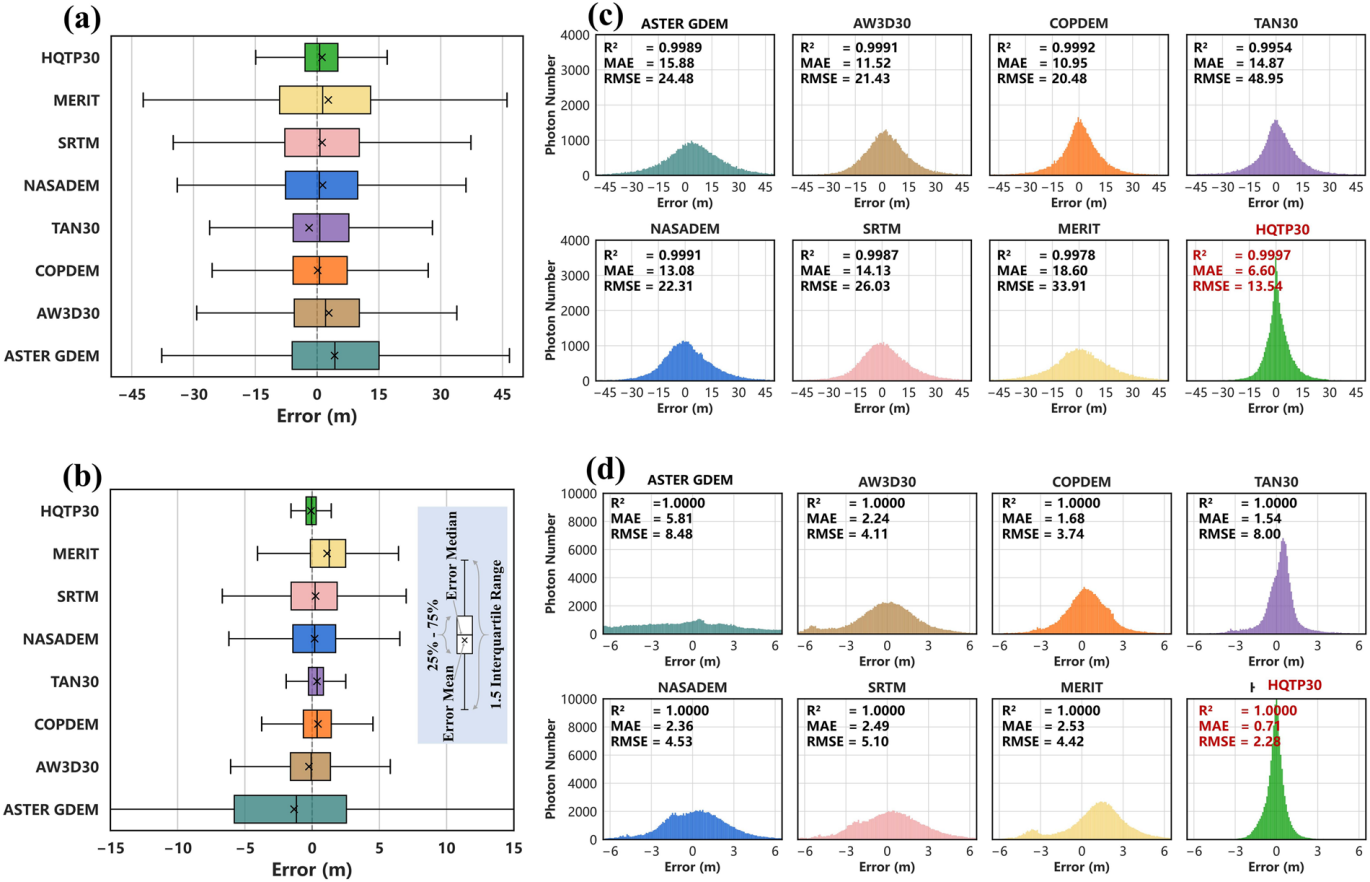
Error Analysis of HQTP30 and Open-access DEMs: (**a**) Error Boxplots in Glacier Regions. (**b**) Error Boxplots in Non-glacier Regions. (**c**) Error Distribution and Statistical Indicators in Glacier Regions. (**d**) Error Distribution and Statistical Indicators in Non-glacier Regions.

Before training the ELF model, 10% of the ATL06 photons (totaling 158,874) and 10% of the ATL08 photons (totaling 119,182) were preserved for validation. Figure 10 presents the error analysis of HQTP30 and open-access DEMs in glacier and non-glacier regions.

In glacier regions, the observed differences between ICESat-2 and DEM are primarily attributed to two factors: (a) the difference in observation times; (b) the inherent errors within the DEM itself. Determining the exact impact of each factor is complex. Nevertheless, given the recognized precision of LiDAR data as an indicator of terrain, we propose that the DEM which most closely aligns with the 2021 LiDAR data provides a more reliable representation of the actual terrain for that year. Conversely, a DEM showing greater deviation with the 2021 LiDAR likely does not reflect the actual terrain accurately.

HQTP30 exhibits the smallest range of errors, while other DEMs, in contrast, display significant error fluctuations and outliers (Fig. 10a). ASTER GDEM significantly reflects the glacier changes between 2000 and 2021, with its elevation average in glacial areas significantly higher than the 2021 level, resulting in substantial positive errors compared to the 2021 ATL06 photons. AW3D30, also derived from optical image, was captured between 2006 and 2011, later than ASTER GDEM; hence its Error Median and Error Mean values are slightly lower than ASTER GDEM. Interestingly, the SRTM data obtained in 2000 is less affected by ice and snow than ASTER GDEM and AW3D30; a similar phenomenon is observed with COPDEM and TAN30 based on TanDEM-X observations. Notably, the recently produced TAN30’s Error Mean is even lower than the 2021 level; whereas COPDEM, observed between 2010–2015, shows Error Mean and Error Median values that are not significantly different from the 2021 levels. It is because ASTER GDEM and AW3D30, as optical observations, cannot penetrate ice and snow, thus reflecting only the pure surface elevation of glaciers; while TAN30, COPDEM, and SRTM, being SAR-based products, can penetrate ice and snow, making their reflection of recent glacial changes less pronounced than ASTER GDEM and AW3D30. Overall, HQTP30 exhibits the highest kurtosis in glacier regions (Fig. 10c), indicating that its error distribution is more concentrated around 0. All open-access DEMs in glacier regions have MAE exceeding 10.95 m and RMSE exceeding 20.48 m. TAN30 even reaches an RMSE of 48.95 m, suggesting the presence of a significant number of outliers in the glacier regions. Conversely, HQTP30 maintains an MAE of 6.60 m and an RMSE of 13.54 m in glacier regions, surpassing all open-access DEMs.

In non-glacier regions, HQTP30 exhibits higher kurtosis and lower skewness (Fig. 10d). HQTP30 has an MAE of 0.71 m and an RMSE of 2.28 m, significantly lower than TAN30’s MAE of 1.54 m and RMSE of 8.00 m, indicating that while TAN30 has a large number of precise pixels, it also has a substantial number of pixels with higher errors, whereas HQTP30 has relatively fewer extreme outliers. Other DEMs exhibit lower kurtosis and higher skewness in both regions, indicating a more dispersed error distribution with a certain degree of overestimation and underestimation.

### Slope-based validation

**Fig. 11:**
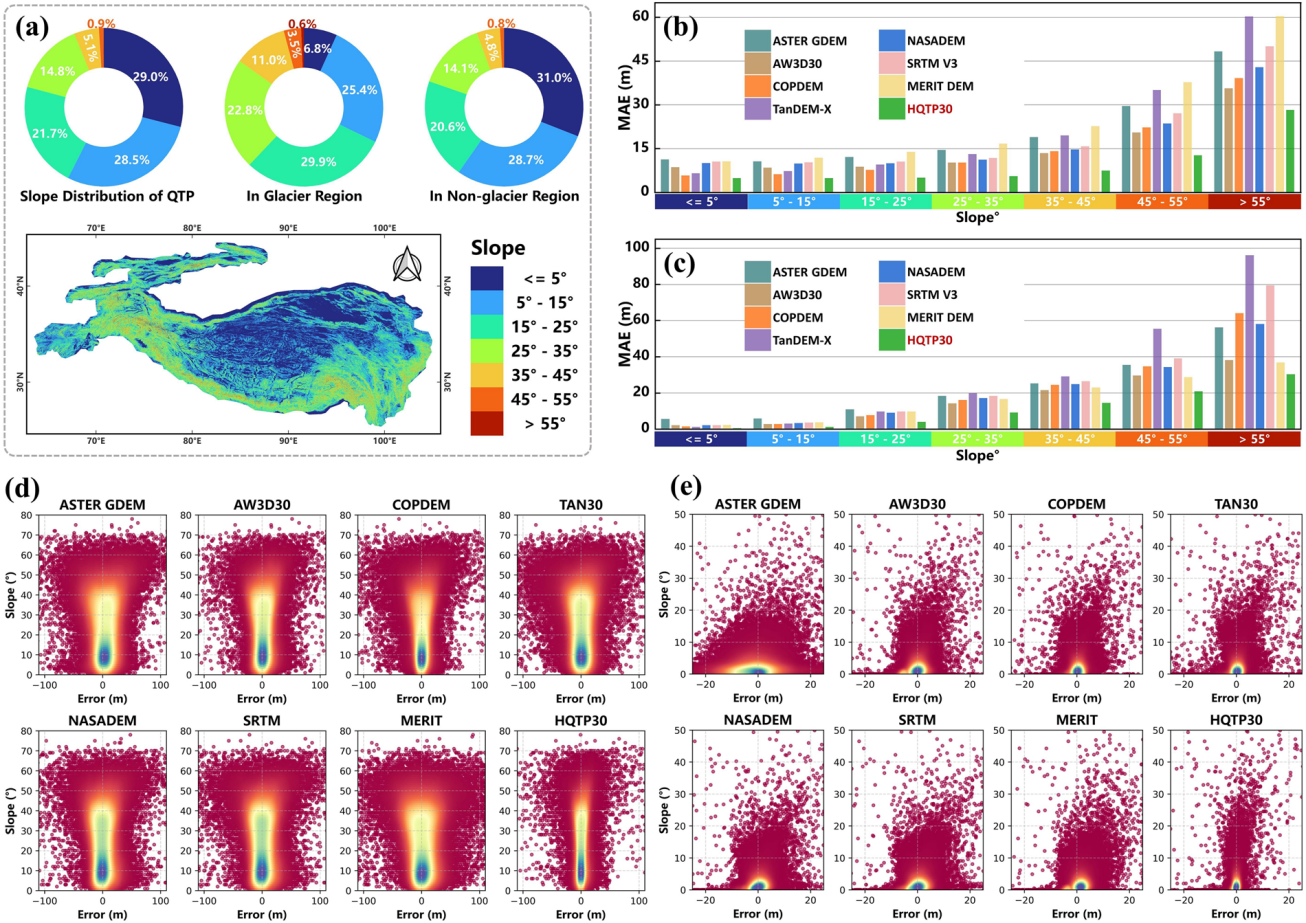
Error Analysis of HQTP30 and Open-access DEMs Across Various Slope Classes: (**a**) Slope Mapping and Percentage Distribution in the QTP. (**b**) MAE in Glacier Regions (**c**) MAE in Non-glacier Regions. (**d**) Kernel Density Estimation of DEM Error vs. Slope in Glacier Regions. (**e**) Kernel Density Estimation of DEM Error vs. Slope in Non-glacier Regions.

Untrained ATL06 and ATL08 validated the accuracy of HQTP30 and open-access DEMs at different slope ranges. The glacier regions of the QTP predominantly feature slopes in the range of 15° to 25° (Fig. 11a). Conversely, non-glacier areas are primarily characterized by slopes below 5°, indicating substantial differences in geomorphological features and necessitating separate discussions. In glacier regions, HQTP30 consistently exhibits the lowest MAE across all slope categories (Fig. 11b), indicating that HQTP30 yields higher accuracy and stability. In contrast, open-access DEMs exhibit significant disparities in MAE across various slope categories, particularly with a noticeable increase in MAE at higher slope levels. In non-glacier regions, HQTP30 maintains the lowest MAE across all other slope categories (Fig. 11c).

Through kernel density estimation, the distribution of DEM errors in different slope ranges can be visually observed (Fig. 11d,e). HQTP30 displayed a more concentrated characteristic across all slope ranges, which indicates that HQTP30 not only possesses lower mean errors but also exhibits more minor error variations and biases. Interestingly, positive errors in open-source DEMs were detected in non-glacial areas (i.e., DEM elevations were higher than those measured by LiDAR), which might be attributed to two main factors: (1) inadequate filtering of vegetation by existing DEMs, leading to an overestimation of terrain height; and (2) LiDAR data collection is confined to June to October, a period less affected by snow cover, whereas the original data for these DEMs were not collected considering periods of maximum snow depth, thereby introducing discrepancies. In summary, HQTP30 performs exceptionally well across various slope levels, affirming the effectiveness and reliability of the HQTP30 generation method.

### Land cover-based validation

**Fig. 12:**
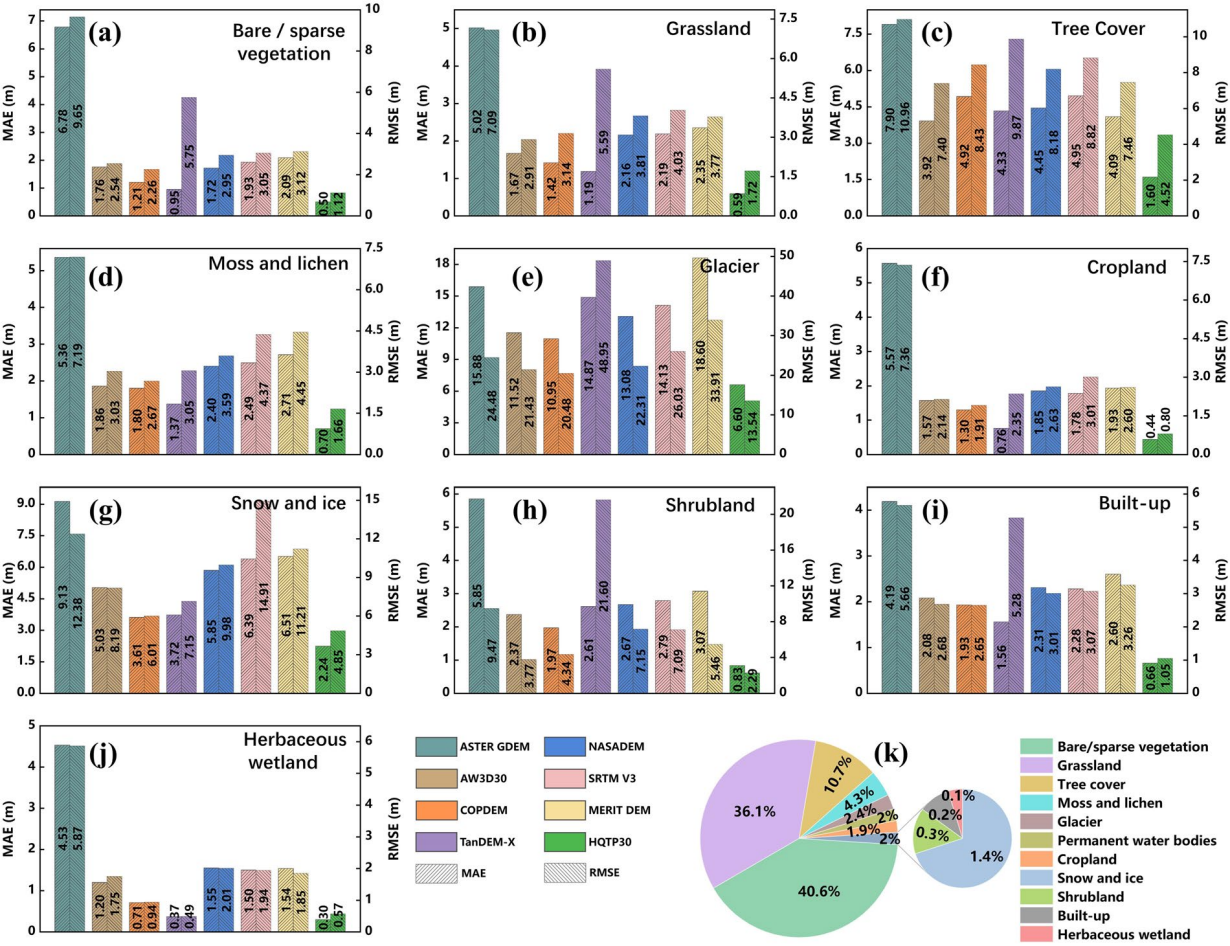
Error Analysis of HQTP30 and Open-Access DEMs Across Diverse Land Cover Types: (**a**–**j**) - MAE and RMSE Distribution for Each DEM Product Across Various Land Cover Types: Bare/Sparse Vegetation, Grassland, Tree Cover, Moss and Lichen, Glacier, Cropland, Snow and Ice (Excluding Glacier Regions), Shrubland, Built-Up, and Herbaceous Wetland. (**k**) Proportion of Land Cover Types in the QTP Based on Merged RGI and WorldCover Datasets.

HQTP30 exhibits significant differences in MAE and RMSE compared to open-access DEMs across different land cover types (Fig. 12). Overall, HQTP30 consistently records the lowest MAE and RMSE, signifying its superior elevation accuracy in QTP. In land cover types such as Bare/sparse vegetation, Grassland, Tree Cover, Moss and lichen, Glacier, Cropland, Snow and Ice (excluding glacier regions), Shrubland, and Built-up, HQTP30’s MAE and RMSE are noticeably lower than those of other DEMs. These land cover types account for 99.9% of the total area in QTP, indicating that HQTP30 maintains a significant accuracy advantage across most of the plateau area.

HQTP30 does not demonstrate a pronounced advantage in MAE and RMSE compared to TAN30 in Herbaceous wetlands, which might be attributed to the relatively scattered distribution of Herbaceous wetlands and the substantial influence of seasonal changes, leading to a certain degree of accuracy reduction in these regions for HQTP30.

### Supplementary information

SUPPLEMENTARY INFORMATION

## Data Availability

The results were generated using Python (version 3.11) and QGIS (version 3.30). Python scripts can be accessed on GitHub at the following repository: https://github.com/ZhangXG-NJU/HQTPDEM.
