# Supplementary material for: Error-Reduced Digital Elevation Model of the Qinghai-Tibet Plateau using ICESat-2 and Fusion Model
Source: Sci Data. 2024 Jun 5;11:588. doi: 10.1038/s41597-024-03428-4 (PMC11153629; doi:10.1038/s41597-024-03428-4)
Supplement: Supplementary file 1 — SUPPLEMENTARY INFORMATION [file 41597_2024_3428_MOESM1_ESM.docx]

# Supplementary File for “Error-Reduced Digital Elevation Model of the Qinghai-Tibet Plateau using ICESat-2 and Fusion Model”

# Authors

Xingang Zhang^1,2^, Shanchuan Guo^1,2,*^, Bo Yuan^1,2^, Haowei Mu^1,2^, Zilong Xia^1,2^, Pengfei Tang^1,2^, Hong Fang^1,2^, Zhuo Wang^3,4^, Peijun Du^1,2^

## Affiliations

1. Key Laboratory for Land Satellite Remote Sensing Applications of Ministry of Natural Resources, Jiangsu Provincial Key Laboratory of Geographic Information Science and Technology, School of Geography and Ocean Science, Nanjing University, Nanjing, China;

2. Jiangsu Center for Collaborative Innovation in Geographical Information Resource Development and Application, Nanjing, China;

3. School of Resource and Environmental Sciences, Wuhan University, Wuhan, China;

4. Faculty of Geomatics, Lanzhou Jiaotong University, Lanzhou, China.

Corresponding author(s): Shanchuan Guo (gsc@nju.edu.cn)

# Section 1. Errors in Open-access DEMs


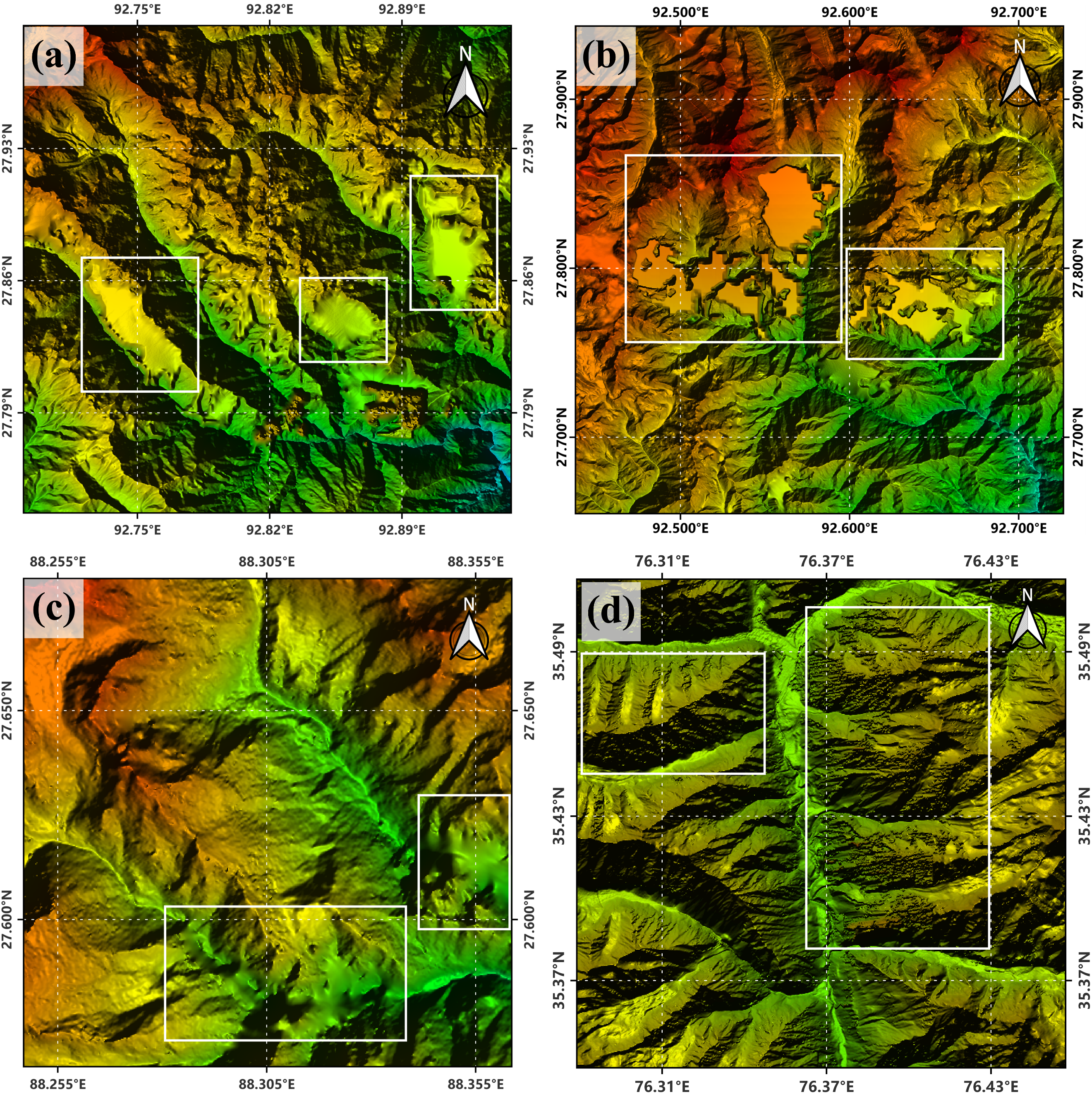


**Figure S1. Noise in the Open-Access DEMs of the QTP. (a) Interpolated Terrain in AW3D30; (b) Faulty Pixels in COPDEM; (c) Topographic Inaccuracies in NASADEM; (d) Significant Terrain Anomalies in TAN30.**

# Section 2. QTP Boundaries

The 2500 m and 3000 m boundaries, extracted from the Earth Topography Five Minute Grid (ETOPO5)[1](#_ENREF_1), demarcate areas with altitudes exceeding 2500 m and 3000 m, respectively. Additionally, the HF Boundary derived from Zhang’s identification of the QTP’s morphological and fundamental features[2](#_ENREF_2). The 2021 Boundary, which accommodates many data sources like Terra Advanced Spaceborne Thermal Emission and Reflection Radiometer (ASTER) Global Digital Elevation Model (GDEM)[3](#_ENREF_3) and Google Earth remote sensing imagery, are also integrated.

# Section 3. Pre-validation using HAGECPD

For the HAGECPD, it’s firstly converted from TOPEX/Poseidon ellipsoid heights ($H_{TOPEX}$)[4](#_ENREF_4) to WGS84 ellipsoid heights ($H_{WGS84}$):

$$\begin{aligned} H_{WGS84}\approx H_{TOPEX}-0.707 m\#\left( 1 \right) \end{aligned}$$

This conversion specifically accounts for the vertical differences, as the horizontal disparities are less than 1 m[5](#_ENREF_5). Subsequently, the $H_{WGS84}$ ellipsoid heights is converted to the EGM2008 geoid heights[6](#_ENREF_6)**.**


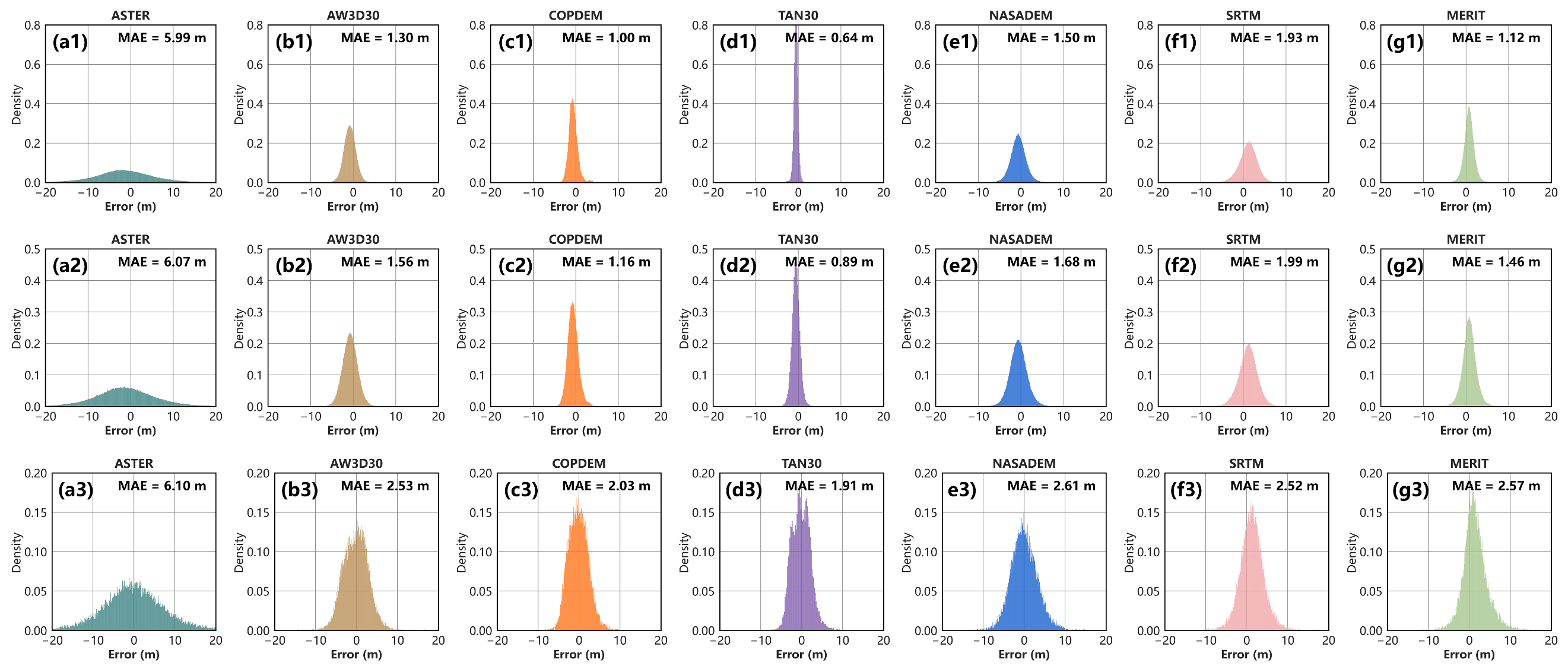


**Figure S2. Pre-validation using HAGECPD. (a1) - (g1) represent assessment results in flat areas (slope < 2°); (a2) - (g2) represent assessment results in hilly areas (2° ≤ slope < 6°); (a3) - (g3) represent assessment results in mountainous areas (6° ≤ slope < 25°).**

We categorized the control points into three groups based on slope: flat (slope < 2°), hilly (2° < slope < 6°), and mountainous (6° < slope < 25°). As Figure S2 shows, the ASTER GDEM lacks satisfactory accuracy in the QTP, exhibiting a Mean Absolute Error (MAE) ranging from 5.99 m to 6.10 m across the three categories, leading to its exclusion from further analysis. The AW3D30 exhibited commendable accuracy, closely following the performance of TAN30 and COPDEM. NASADEM, an enhanced version of SRTM V3, incorporates multiple remote sensing datasets to enhance data quality, and therefore, we included it while excluding SRTM V3. Both COPDEM and TAN30 are derived from TanDEM-X observation mission, while utilize distinct processing techniques. TAN30 demonstrated superior performance across all terrain categories, achieving the lowest MAE, with COPDEM's accuracy closely following. TAN30 and COPDEM are considered complementary rather than mere alternatives. Fig. S3 presents two examples, where COPDEM is better in Area 1, while TAN30 is better in Area 2.


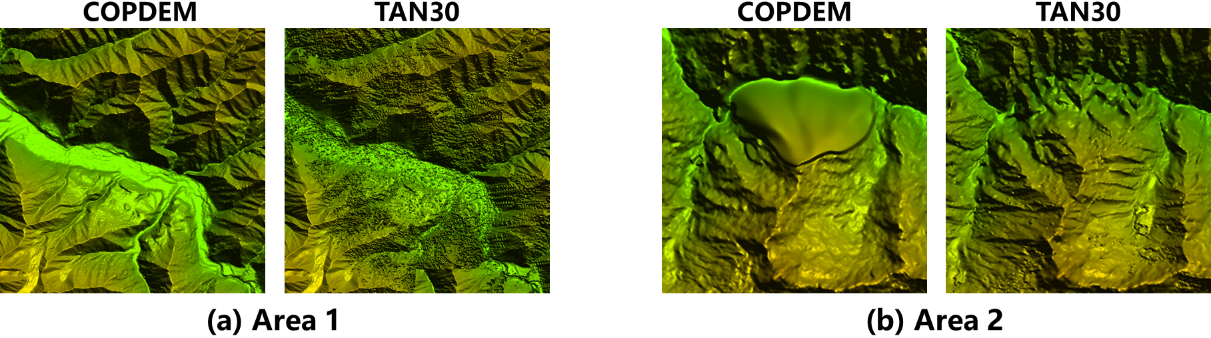


**Figure S3. COPDEM and TAN30 in two areas.**

Please note that HAGECPD covers elevation control points within land areas with slopes less than 25° and does not encompass extremely rugged and steep topographical regions. Nonetheless, it does provide a valuable perspective for assessing DEM accuracy within this specific region.

# Section 4. Pre-validation using ATL08 for Non-Glacier Regions

Following photon parameter extraction and multi-temporal data integration filtering of ATL08, it was employed for DEM pre-validation for QTP's stable regions. The error distribution and quantitative metrics (Fig. S4) indicated that, for glacier regions, the MAE for 2021, in ascending order, was as follows: TAN30 (1.54m), COPDEM (1.68 m), AW3D30 (2.24 m), NASADEM (2.36 m), SRTM (2.49 m), MERIT DEM (2.53 m), and ASTER GDEM (5.81 m).


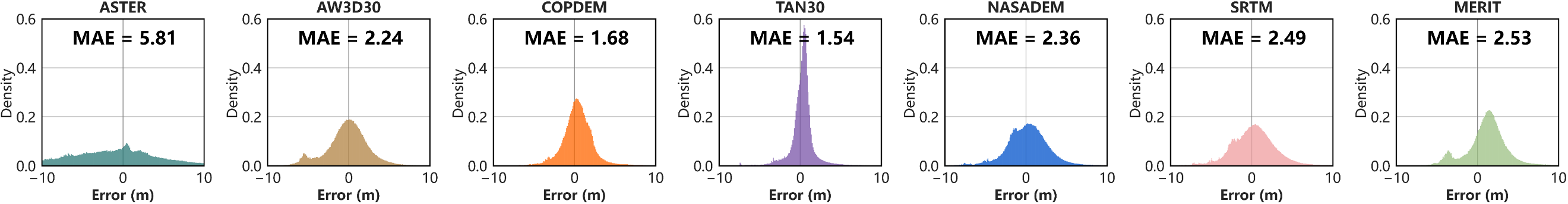


**Figure S4. Pre-validation using ATL08.**

# Section 5. Elevation Reference Harmonization

For TanDEM-X, the conversion from the ellipsoidal h eight based on WGS84 ($H_{WGS84}$) to the orthometric height based on EGM2008 ($H_{EGM2008}$) is executed as follows:

$$\begin{aligned} H_{EGM2008}=H_{WGS84}-{NGH}_{EGM2008}\#\left( 2 \right) \end{aligned}$$

In this equation, ${NGH}_{EGM2008}$ represents the geoid undulation between the equipotential surface of EGM2008 and the WGS84 ellipsoid. For AW3D30 and NASADEM, the original orthometric heights based on EGM96 ($H_{EGM96}$) are converted to $H_{EGM2008}$:

$$\begin{aligned} H_{EGM2008}=H_{EGM96}+{NGH}_{EGM96}-{NGH}_{EGM2008}\#\left( 3 \right) \end{aligned}$$

Here, ${NGH}_{EGM96}$ signifies the geoid undulation between the equipotential surface of EGM96 and the WGS84 ellipsoid. Notably, COPDEM, already based on EGM2008 geoid heights, requires no elevation conversion. The values of ${NGH}_{EGM96}$ and ${NGH}_{EGM2008}$ can be accessed at [www.agisoft.com/downloads/geoids/](http://www.agisoft.com/downloads/geoids/).

# Section 6. Model Interpretation

SHapley Additive Explanations (SHAP) values are a crucial method for interpreting machine learning model predictions[7](#_ENREF_7). SHAP values calculate the marginal contribution of each feature to the model’s prediction. By taking the average absolute values of the SHAP values, the average influence of each feature on the overall dataset can be obtained. Figure S5 presents the average SHAP values of various features when predicting elevation values using different machine learning regression models (Random Forest, ExtraTrees, XGBoost) in different regions (glacier and non-glacier).

In glacier regions (Figure S5a), both the SRTM DEM and TAN DEM are shown to have high importance across all three models, with values such as 281.619, 149.836, 202.677 (SRTM DEM) and 193.250, 168.749, 390.785 (TAN DEM). The importance scores for SRTM NNA and TAN NNA vary across the models. For example, SRTM NNA has values of 53.827 in Random Forest, 144.840 in ExtraTrees, and 9.049 in XGBoost, while TAN NNA has values of 77.501, 123.540, and 7.036, respectively. Snow Cover shows a high importance score of 17.211 in the ExtraTrees but relatively low importance in the Random Forest and XGBoost (1.286 and 4.372, respectively).

In non-glacier regions (Figure S5b), COP DEM demonstrates extremely high importance in the XGBoost, with a value of 851.565, significantly higher compared to its importance in Random Forest (222.199) and ExtraTrees (215.089). NASA NNA shows significant importance across multiple models, with values such as 42.006 in XGBoost and relatively high scores in other models (e.g., 24.271 in ExtraTrees). Both AW3D DEM and NASA DEM have high importance scores in several models. For instance, AW3D DEM scores 173.193 in Random Forest, 207.886 in ExtraTrees, and 131.098 in XGBoost. NASA DEM shows 190.997 in Random Forest, 190.909 in ExtraTrees, and 2.647 in XGBoost.


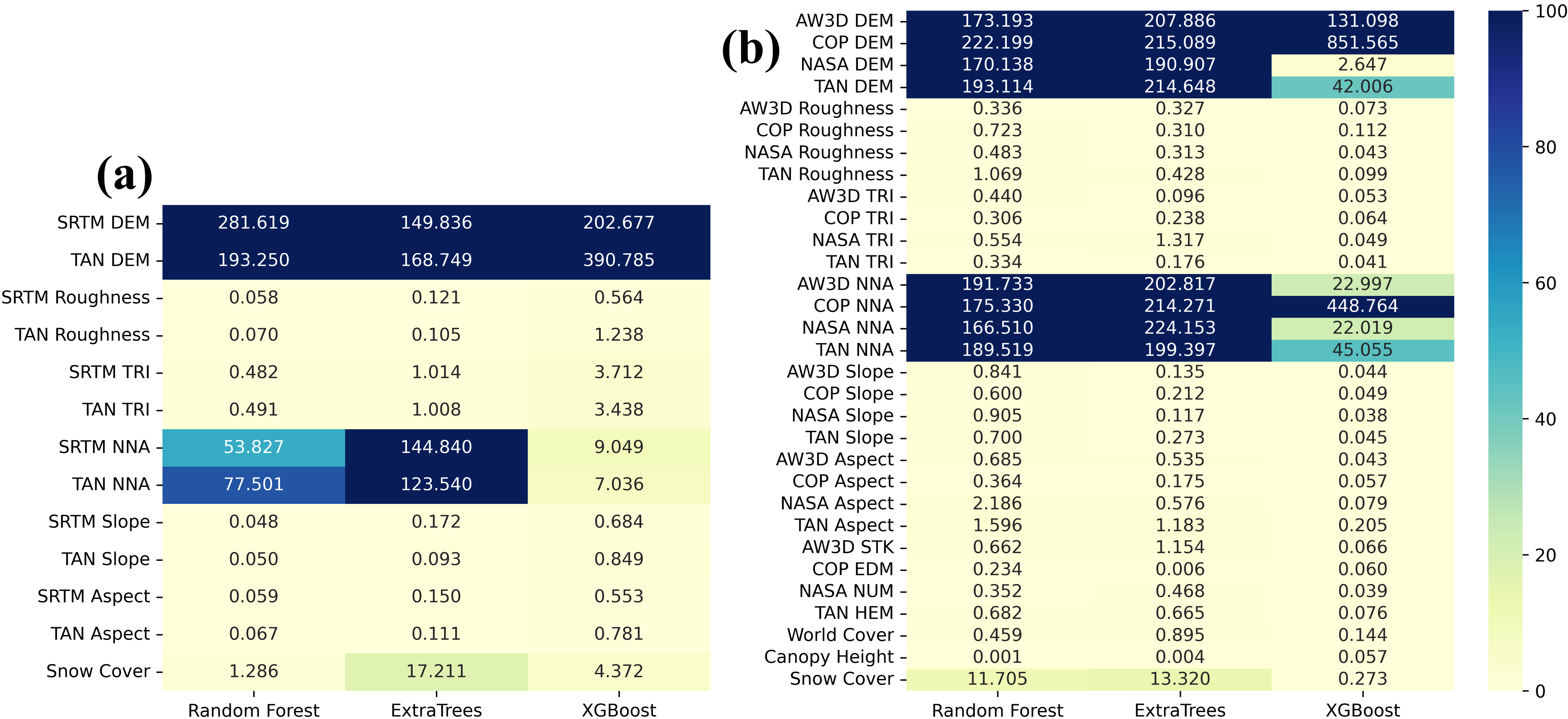


**Figure S5. SHAP value of each feature for Random Forest, ExtraTrees, and XGBoost. (a) In Glacier Regions; (b) in Non-glacier Regions.**

Noted that although terrain features (e.g., NNA, aspect, slope) contribute less to reconstruction results, they are crucial for improving accuracy (Figure S6). The Mean Absolute Error (MAE) decrease as the number of features increases (the values on the horizontal axis represent new features added on top of the features to their left). Initially, the MAE decreases markedly as more features are added, then the reduction rate slows, suggesting that while the initial inclusion of new features significantly improves model accuracy, the marginal benefit of additional features diminishes over time. Nonetheless, even after reaching a certain number of features, introducing new features continues to improve the accuracy of the DEM. Therefore, in practical applications, it is essential to select and incorporate a variety of effective features to enhance DEM prediction accuracy. Note that to reduce the risk of overfitting, the depth of the tree is recommended to be smaller than the dimension of the features.


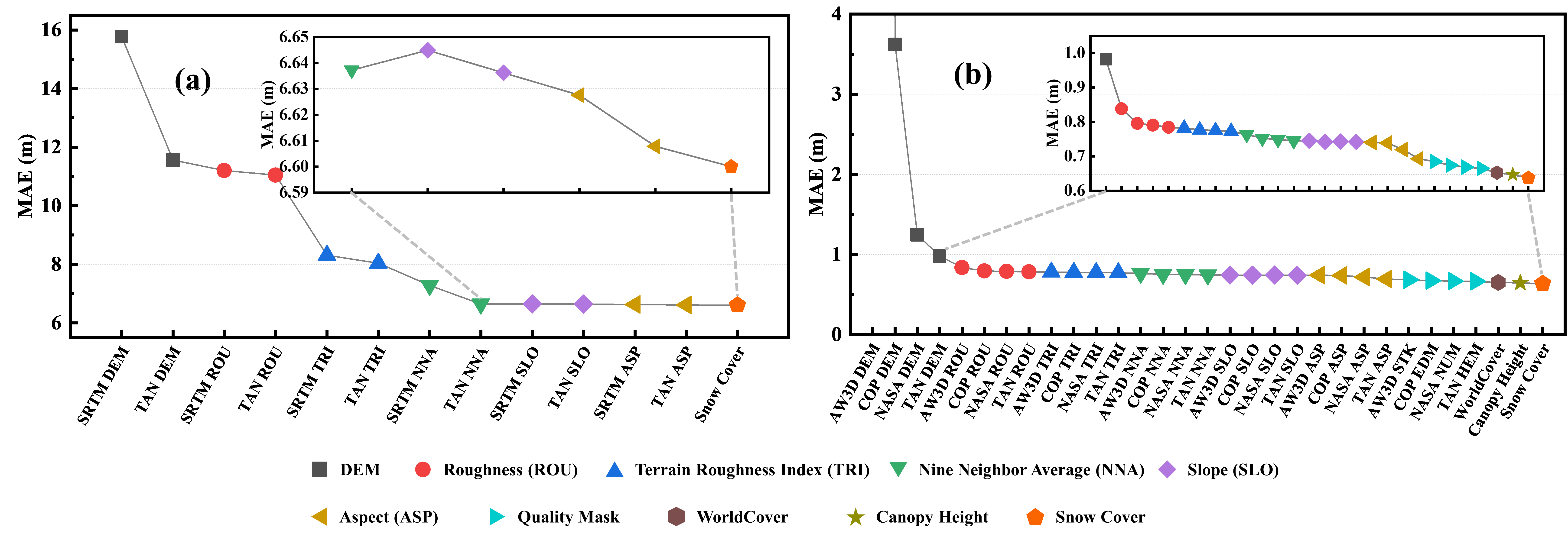


**Figure S6. MAE changes with the number of features increase. (a) In Glacier Regions; (b) in non-glacier regions.**

# Section 7. Comparison with High-resolution DEMs


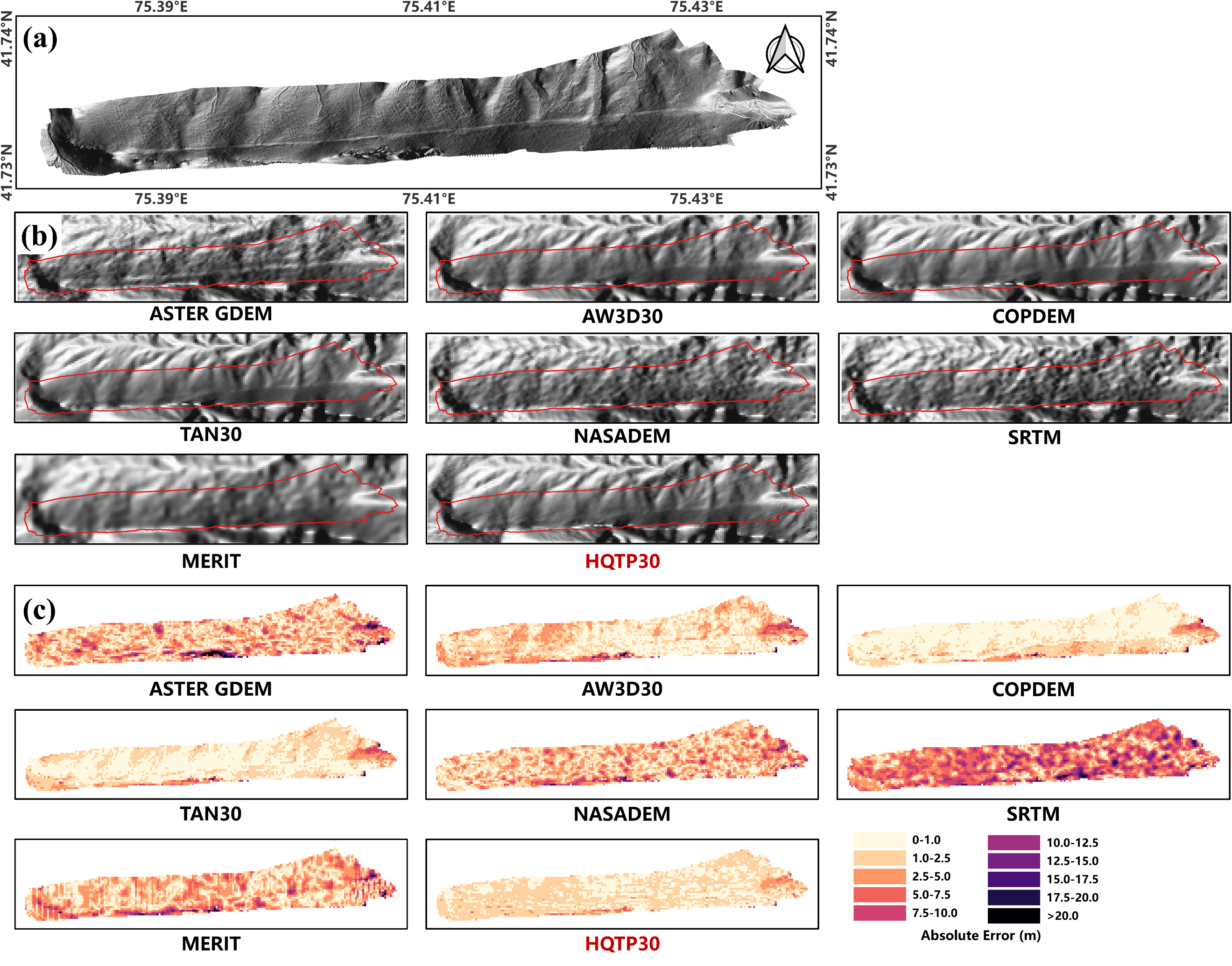


**Figure S7. Comparison of UAV-derived DEM, HQTP30, and Open-Access DEMs in Fault South of Song Kul, Kyrgyzstan. (a) UAV-derived DEM; (b) Terrain Rendering Comparison; (c) Error Details.**


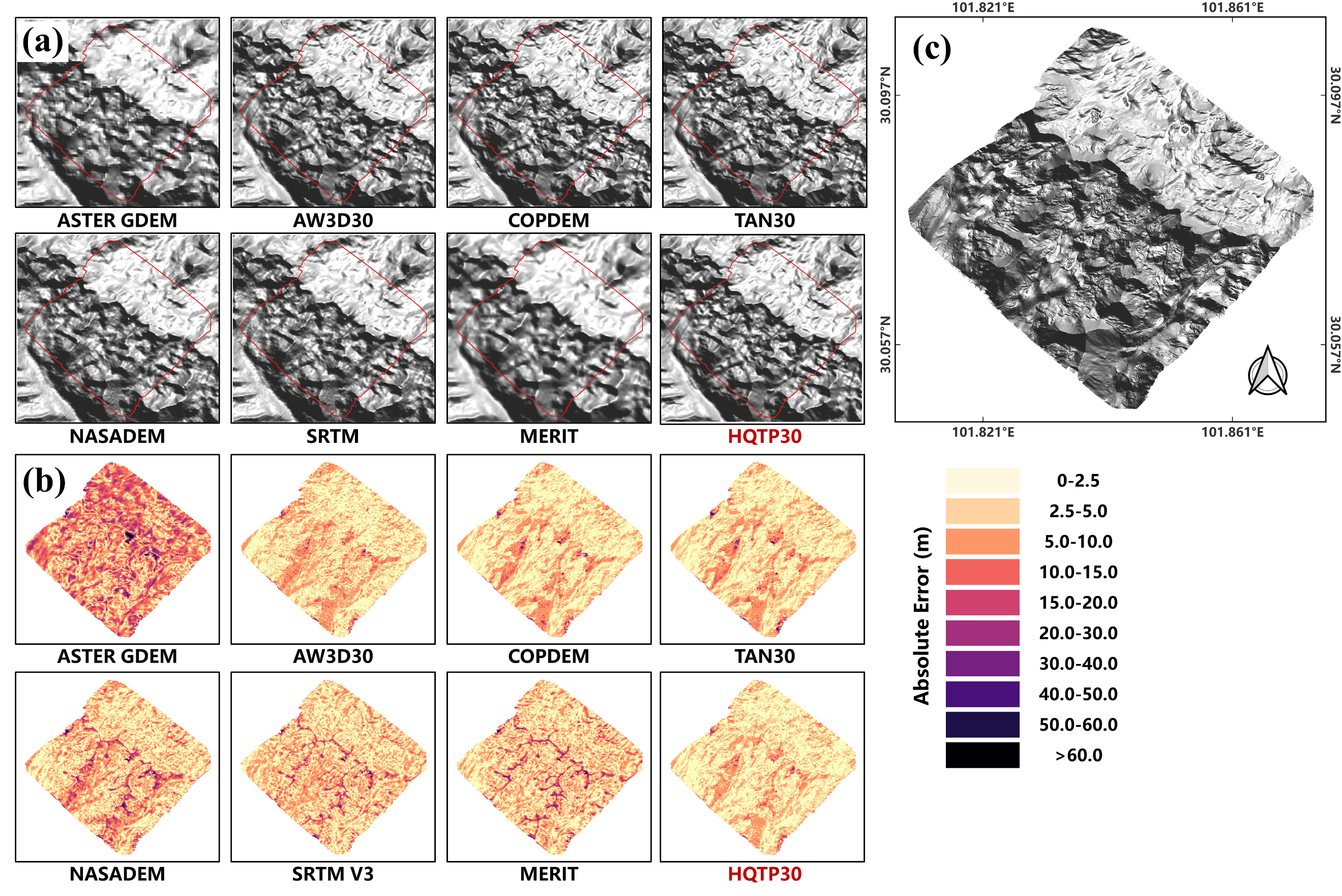


**Figure S8. Comparison of UAV-derived DEM, HQTP30, and Open-Access DEMs in Zheduoshan Mountain. (a) UAV-derived DEM; (b) Terrain Rendering Comparison; (c) Error Details.**

**References**

1 Center, N. G. D. (National Geophysical Data Center NOAA Silver Spring, MA, USA, 1993).

2 Zhang, Y., Li, B., Liu, L. & Zheng, D. Redetermine the region and boundaries of Tibetan Plateau. *GEOGRAPHICAL RESEARCH* **40**, 1543-1553 (2021).

3 Yamaguchi, Y., Kahle, A. B., Tsu, H., Kawakami, T. & Pniel, M. Overview of advanced spaceborne thermal emission and reflection radiometer (ASTER). *IEEE Trans. Geosci. Remote Sens.* **36**, 1062-1071 (1998).

4 Fu, L. L. *et al.* Vol. 99 24369-24381 (Wiley Online Library, 1994).

5 Yue, L. *et al.* High-quality seamless DEM generation blending SRTM-1, ASTER GDEM v2 and ICESat/GLAS observations. *ISPRS J. Photogramm. Remote Sens.* **123**, 20-34 (2017).

6 Pavlis, N. K., Holmes, S. A., Kenyon, S. C. & Factor, J. K. The development and evaluation of the Earth Gravitational Model 2008 (EGM2008). *J. Geophys. Res.: Solid Earth* **117**, doi:<https://doi.org/10.1029/2011JB008916> (2012).

7 Antwarg, L., Miller, R. M., Shapira, B. & Rokach, L. Explaining anomalies detected by autoencoders using Shapley Additive Explanations. *Expert Syst. Appl.* **186**, 115736 (2021).
